# Photocrosslinkable lung dECM hydrogels promote stiffness-dependent lung cancer growth and chemoresistance

**DOI:** 10.1016/j.mtbio.2026.102838

**Published:** 2026-01-24

**Authors:** Luke Hipwood, Minne Dekker, Dietmar W. Hutmacher, Christoph Meinert, Jacqui A. McGovern

**Affiliations:** a Faculty of Health, School of Biomedical Sciences, Queensland University of Technology, Brisbane, QLD, 4000, Australia; b Centre for Biomedical Technologies, Queensland University of Technology, Brisbane, QLD, 4059, Australia; c Gelomics Pty Ltd, Brisbane, QLD, 4059, Australia; d Max Planck Queensland Centre (MPQC) for the Materials Science of Extracellular Matrices, Queensland University of Technology, Brisbane, QLD, 4059, Australia; e Australian Research Council (ARC) Training Centre for Multiscale 3D Imaging, Modelling and Manufacturing (M3D Innovation), Queensland University of Technology, Brisbane, QLD, 4059, Australia; f ARC Training Centre for Cell and Tissue Engineering Technologies (CTET), QUT, Brisbane, QLD, 4059, Australia; g Translational Research Institute, Queensland University of Technology, Woolloongabba, Q:D, 4102, Australia

**Keywords:** decellularized extracellular matrix, lung cancer, hydrogel stiffness, Tumour microenvironment, 3D cell culture, In vitro model, Biomaterial

## Abstract

Decellularized extracellular matrices (dECMs) are promising biomaterials for generating tissue-specific *in vitro* models due to their organotypic extracellular matrix (ECM) protein profiles compared to natural and synthetic alternatives. However, most dECM-based hydrogels rely on collagen fibrillogenesis, resulting in limited mechanical tuneability and cell instructivity. Here, we developed LungMA, a photocrosslinkable, methacrylated lung dECM hydrogel engineered for precise stiffness modulation and tissue-specific lung cancer modelling. The decellularization process removed >99 % of native DNA, ensuring minimal cellular remnants while preserving key ECM components including laminin-332, collagen VI, and heparan sulfate proteoglycan 2 (HSPG2). Methacrylation and photoinitiation enabled formation of stable LungMA hydrogels with tunable stiffnesses ranging from 1 kPa (healthy lung) to 4 kPa (fibrotic lung).

Using A549 non-small-cell lung cancer (NSCLC) cells, we demonstrated that matrix composition and stiffness influenced cell morphology, proliferation, and drug response. Soft LungMA (1 kPa) promoted motile, sheet-like cellular organization, whereas stiff LungMA (>4 kPa) induced compact spheroids associated with chemoresistance. Increasing matrix stiffness resulted in an increase in doxorubicin IC_50_ from 0.40 μM (soft LungMA) to 1.23 μM (stiff LungMA), and cisplatin IC_50_ from 0.03 μM to 8.34 μM, reflecting clinical observations where fibrosis correlates with poor prognosis.

In contrast, gelatin methacryloyl (GelMA) and basement membrane extract (BME)-based hydrogels failed to induce these stiffness-dependent effects during cisplatin treatment underscoring the instructive role of lung-specific ECM components and matrix stiffness on chemotherapeutic outcomes.

LungMA provides a physiologically relevant, mechanically tunable, lung-specific platform that replicates *in vivo*-like cancer phenotypes and drug responses. This work supports the application of LungMA for oncology research, disease modelling, and high-throughput drug screening as a clinically relevant, non-animal alternative for lung cancer studies.

## Highlights

• LungMA is a photocrosslinkable lung dECM hydrogel with tunable stiffness and high cellular compatibility.
• Matrix stiffness (1–4+ kPa) influenced A549 cell organisation and spheroid morphology.
• Soft LungMA induced sheet-like growth while stiff LungMA promoted compact spheroid formation.
• Doxorubicin resistance increased with stiffness: IC_50_ rose from 0.40 μM to 1.23 μM in LungMA.
• GelMA and BME did not reproduce the stiffness-dependent effects on NSCLC behaviour observed in LungMA.

## Graphical abstract

**Figure:**
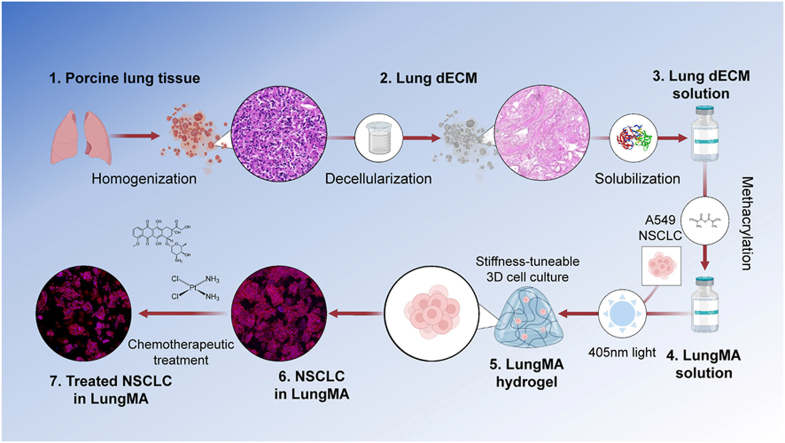

## 1 Introduction

Lung-associated diseases are among the leading causes of deaths globally, with conditions such as primary lung cancer representing over 1.8 million mortalities annually [1]. With the advent of the Food and Drug Administration Modernization Act 2.0 in 2022, advances in functional drug testing methods have become less constrained, therefore promoting the development of more innovative, human-relevant, and effective *in vitro* models that could be used for preventing and treating diseases of the lung, such as lung cancer [2,3]. Of note are *in vitro* models that incorporate decellularized extracellular matrices (dECMs) harvested from specific organs and tissues, providing tissue-specific microenvironments for cell culture and disease modelling. As a result of their inherent tissue-specific protein profiles, previous reports utilizing these biomaterials derived from organs such as the liver [4,5], heart [[6], [7], [8]], kidney [[9], [10], [11]], intestines [[12], [13], [14]], bone [[15], [16], [17]], and lung [18,19], have shown such materials to replicate organotypic cell culture characteristics better and enhance physiological relevance compared to other models. This superiority has been demonstrated by increased albumin production in liver dECMs compared to 2D cell cultures [4,5], BME-free generation of intestinal organoids in intestine dECM, and improved osteogenic differentiation of stem cells cultured in bone dECMs, to name a few [12,13,15,16]. Regarding lung dECMs, they have been used for various lung-specific studies, such as the formation of differentiated epithelial layers using fibroblasts and epithelial cells, *in vitro* preservation of alveolar type 2 phenotypes in alveolar epithelial cells, and determining the effect of idiopathic pulmonary fibrosis (IPF) on cell orientation and ECM production. [[17], [18], [19], [20], [21], [22], [23]].

However, most current dECM-based hydrogels suffer from poor mechanical control, as they either function as supplements within bulk hydrogels or rely on collagen fibrillogenesis, resulting in inconsistent gelation, variable porosity, and non-physiological stiffness (Table S12) [[24], [25], [26], [27], [28]]. This is particularly problematic in lung cancer studies, where the tumour microenvironment often exhibits altered stiffness, driving disease progression and chemoresistance. Current systems including commercially available lung dECMs, rarely achieve reproducible and tunable mechanical properties across physiological and fibrotic stiffness ranges (Table S12). To address these limitations, chemical functionalization with thiol (-SH), norbornene (-NB), or methacryloyl (-MA) groups has been employed to enable covalent crosslinking and tunable mechanical properties of dECMs [[29], [30], [31], [32], [33]]. Among these, methacrylated dECM (dECM-MA) hydrogels have gained traction due to their ability to form stable, photocrosslinked matrices under cell-compatible visible light, allowing precise tuning of stiffness and crosslinking density [34]. These hydrogels are particularly valuable for studying fibrosis-related diseases, as they can be tailored to match native and fibrotic organ stiffnesses, providing a more physiologically relevant environment for long-term cell culture [35,36]. Despite these advances, many existing lung dECM studies have focused on regenerative applications or lung cancer studies in un-functionalized material, with the effect of stiffness-modulation on lung cancer, and specifically non-small lung cancer (NSCLC) response to chemotherapy still being relatively unexplored [[37], [38], [39], [40], [41], [42], [43], [44], [45]]. Previous studies have utilized stiffness-tunable unfunctionalized lung, and thiolated lung dECMs for the investigation of IPF [[46], [47], [48]]. Additionally, methacrylated lung (LungMA) hydrogels have been used for culture and treatment of small-cell lung cancer (SCLC), however, LungMA hydrogels have not yet been utilized for culture and treatment of NSCLC [49,50].

NSCLC is the most common type of lung cancer, comprising roughly 85 % of cases, and is defined as any type of epithelial lung cancer other than SCLC [[51], [52], [53], [54]]. NSCLC onset is predominantly associated with inhalation-based risk factors such as smoking, occupational exposure to particles such as asbestos, and chronic environmental exposure to air pollution, which contribute towards oncogenic mutations in genes such as TP53 and KRAS [55,56], and increase in adverse pathologies such as elevated lung tissue fibrosis [[57], [58], [59]]. Fibrotic lung stiffness can contribute towards chemoresistance through impairment of T-cell infiltration, matrix remodelling, and pro-tumoral stiffness-dependent cell-ECM signaling pathways [[60], [61], [62], [63]]. Despite lung stiffness playing a crucial role in the progression and outcomes of NSCLC, there are currently no reports detailing the effect of matrix stiffness on NSCLC response to treatment when cells are cultured in LungMA hydrogels of healthy lung representative (1 kPa) or fibrotic lung representative (>4 kPa) matrices. Furthermore, *in vitro* lung cancer treatment outcomes achieved in stiffness-matched lung dECM-based materials have not yet been directly compared to non-lung-specific methacrylated hydrogels, such as gelatin methacryloyl (GelMA).

Here, we report the synthesis, characterization and application of methacrylated LungMA hydrogels for 3D culture and chemotherapeutic testing of NSCLC. Prepared through decellularization, methacrylation, and photocrosslinking, LungMA hydrogels retained a high proportion of the native tissue's protein profile. They formed hydrogels with tunable stiffnesses representative of healthy (∼1 kPa) and fibrotic (>4 kPa) lung tissue. Compared to GelMA and BME-based 3D culture systems, LungMA hydrogels supported stiffness-dependent tumour growth and response to cisplatin treatment, recapitulating trends in clinical lung cancer outcomes in fibrotic environments^.^ [37,38] Our findings suggest that cell-ECM interactions facilitated by ECM matrisome composition and stiffness, are critical for *in vitro* cell culture and chemotherapeutic outcomes, and demonstrate the utility of LungMA hydrogels for *in vitro*, tissue-specific 3D cell culture compared to alternative hydrogel systems. Overall, this work advances our understanding of disease mechanobiology by demonstrating matrix mediated resistance to chemotherapy.

## 2 Results and discussion

### 2.1 Lung decellularization

First, we systematically optimized lung decellularization by testing various detergent, salt, and combination protocols to identify conditions that maximized DNA and nuclei removal while preserving sulphated glycosaminoglycans (sGAGs) and ECM integrity. We selected Triton X-100 (non-ionic), sodium laureth sulfate (SLES) (anionic), and CHAPS (zwitterionic) based on their ionic properties and prior use in dECM preparation [[64], [65], [66], [67], [68], [69]]. It was hypothesized that sequential detergent-salt treatment would be more effective than detergent-only methods, as NaCl aids in both decellularization and debris removal [69]. Additionally, we expected SLES to decellularize lung tissue faster but at the cost of more significant ECM degradation, consistent with prior studies on SLES and SDS-induced ECM damage [[66], [67], [68]].

**Fig. 1:**
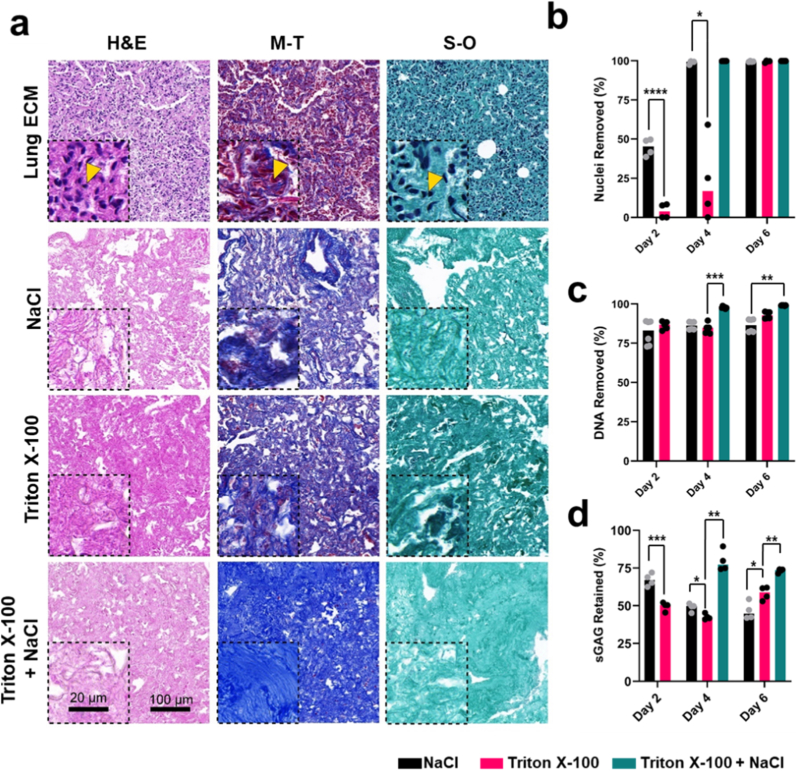
**Decellularization of porcine lung tissue using detergent and hypertonic solutions. (A)** Histological staining of lung tissues on day 6 of decellularization. Hematoxylin and eosin (H&E) reveals nuclei (purple) and background ECM (pink). Masson's trichrome (M–T) staining of tissue nuclei (dark brown), collagen (blue) and cytoplasm (red). Safranin-O (S–O) staining of nuclei (blue) and ECM (green). Yellow arrows = nuclei. Scale = 100 μm, inset scale = 20 μm. Histological images are representative n = 4 replicate images that were imaged via Caseviewer. **(B)** Nuclei removed over time, determined by quantifying tissue nuclei normalized to tissue area in region of interest (ROI) using Image J software. N = 1. n = 4. **(C)** Lung dECM DNA content over time was quantified by Quant-iT™ PicoGreen™ assay and normalized to sample weight. N – 1. n = 6. **(D)** The concentration of sulphated glycosaminoglycans (sGAGs) in lung dECMs over time was quantified by DMMB assay and normalized to the sample weight. N = 1. n = 4. Mann Whitney tests were used to analyze day 2 samples, while non-parametric ANOVA tests were used to analyze day 4 and day 6 samples. All data is represented as mean ± SD. ∗*p* < 0.1, ∗∗*p* < 0.01, ∗∗∗*p* < 0.001, ∗∗∗∗*p* < 0.0001.

Masson's trichrome staining (Fig. 1A) confirmed that Triton X-100 + NaCl retained ECM components while entirely removing cellular material, whereas Triton X-100 or NaCl alone left cytoplasmic remnants. Quantification of nuclei in histological sections (Fig. 1B), DNA content (Fig. 1C), and sGAG retention (Fig. 1D) further supported these findings, demonstrating >99 % DNA and nuclei removal with preserved sGAG content. Similarly, CHAPS alone failed to decellularize lung tissue fully, but CHAPS + NaCl resulted in complete decellularization (Fig. S1), reinforcing the effectiveness of sequential detergent-salt protocols. SLES was the most effective standalone detergent for decellularization, achieving faster cellular removal than Triton X-100 and CHAPS (Fig. S1). However, SLES-treated dECMs exhibited weak ECM staining, collagen fiber distribution (Fig. S1A), and cytotoxicity in initial cytocompatibility studies (Fig. S3), likely due to residual SLES retention within the ECM [28]. These findings suggest that SLES remains embedded in the lung ECM, leading to cytotoxic effects, making it unsuitable for lung dECM preparation and cell culture applications. Increasing NaCl concentration beyond 1M did not enhance decellularization compared to 1M NaCl or sequential detergent-salt protocols (Fig. S2). Among the tested methods, Triton X-100 + NaCl (Fig. 1) and CHAPS + NaCl (Fig. S1) produced dECMs with the most favorable characteristics for *in vitro* applications, exhibiting high histological ECM retention and efficient removal of cellular and nucleic material. Based on these findings, we selected Triton X-100 + NaCl as the optimal decellularization method for downstream experiments.

### 2.2 Proteomic analyzes of porcine lung ECM and dECM reveal a conserved matrisome post-decellularization

After identifying Triton X-100 + NaCl as the optimal lung decellularization method, we analyzed the matrisome composition of both lung ECM and Triton X-100 + NaCl decellularized dECM to assess protein retention post-decellularization and compared these results to a commercial BME, commonly used as a matrix for 3D cell culture. Given that the matrisome profile of cell culture matrices influences mechanical properties, cell viability, and *in vitro* cell behavior [5,[70], [71], [72], [73], [74]], we hypothesized that lung dECM would possess a proteomic profile similar to that of lung ECM, therefore creating a more physiologically relevant microenvironment for NSCLC investigation than BME.

**Fig. 2:**
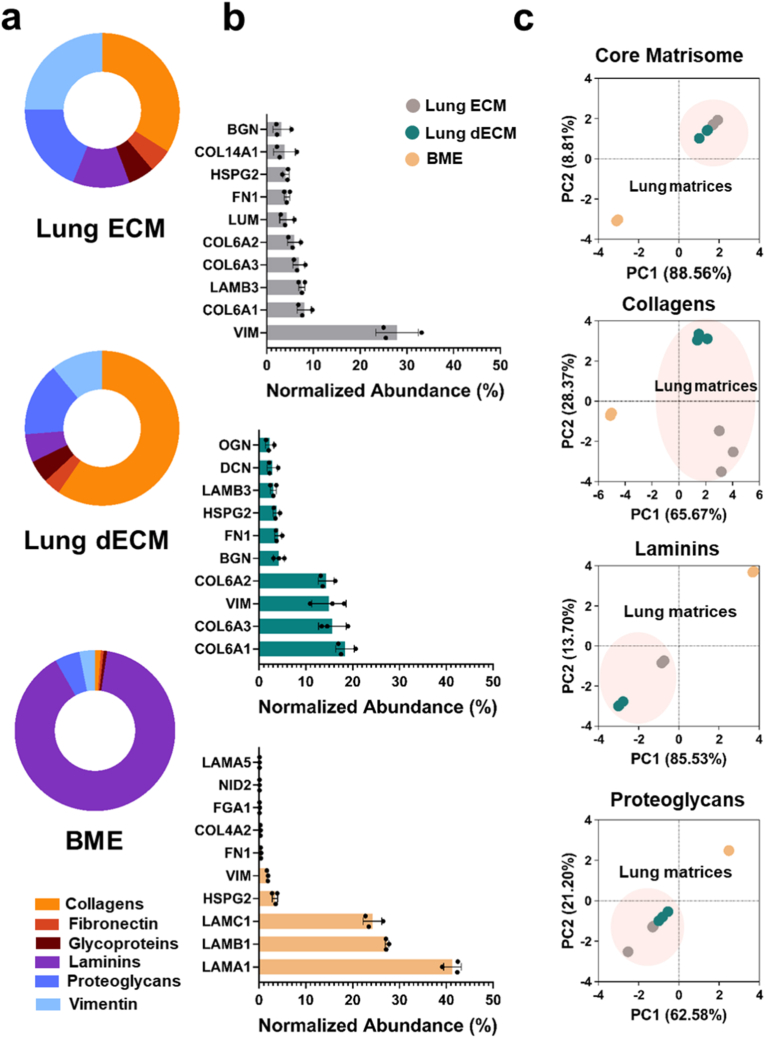
**Proteomic analyzes of porcine lung ECM, dECM, and BME reveals conservation of lung ECM core matrisome proteins post-decellularization.** The abundance of proteins was normalized against total protein abundance within each group to determine protein distribution and rank. Principal core analyzes (PCA) of protein profiles of lung ECM, dECM, and BME samples displayed a conservation of proteins between the lung ECM and dECM groups. **(A)** Distribution of core matrisome proteins in lung ECM, lung dECM and BME. **(B)** Normalized abundance of the 10 most abundant core matrisome proteins within lung ECM, lung dECM, and BME. N = 1. n = 3. All data is represented as mean ± SD. **(C)** PCA of core matrisome proteins, collagens, laminins, and proteoglycans. N = 1. n = 3.

Principal component analysis (PCA) confirmed that the core matrisome of lung ECM remained broadly conserved post-decellularization. In contrast, BME exhibited a distinct matrisome profile, differing significantly from both lung ECM and dECM (Fig. 2A and B). The lung ECM and dECM were majorly constituted of collagens (32.21 % of lung ECM core matrisome proteins and 54.66 % of lung dECM core matrisome proteins) (Table S4), with collagen type VI being the most abundant collagen in both the ECM and dECM groups (Table S5). Collagen VI comprises three distinct polypeptide chains: α1, α2, and α3, which assemble into heterotrimeric microfilaments. These networks are predominantly found in the basement membrane, providing structural support and regulating cellular behavior by facilitating cell adhesion, proliferation, and migration [[73], [74], [75]]. The abundance of collagen VI may influence the tumor microenvironment (TME), as previous studies have presented associations between elevated collagen VI expression and NSCLC development, proliferation and bone metastasis [76,77]. Furthermore, PCA revealed a difference in the collagen composition of lung samples compared to BME, with the most abundant collagen in the BME being collagen type IV (Table S5). Despite being a different collagen type, collagen IV is similar to collagen VI in that both are localized within the lung basement membrane [59,78]

Following collagen, the most abundant ECM protein we identified in the lung ECM and dECM samples was vimentin (Fig. 2B and Table S4). Vimentin was also present in the BME; however, it only accounted for 1.85 ± 0.13 % of the core matrisome. Vimentin appears in various tissues both in intracellular and extracellular compartments. It plays crucial roles in disease state progression, particularly in epithelial-to-mesenchymal transition (EMT), cancer metastasis, and tissue remodelling [79]. Importantly, Beijnum et al. found that extracellular vimentin could mimic the function of vascular endothelial growth factor (VEGF) through activation of VEGF receptor 2, thereby promoting angiogenesis [80]. Therefore, the presence of vimentin within the lung ECM and dECM may influence the ability to promote EMT and angiogenesis.

Like collagen type VI, laminins are major structural components of the basement membrane and are integral for matrisome functions such as cell adhesion, growth and proliferation [81,82]. Laminins are believed to be one of the main drivers for *in vitro* organoid formation, with the ability for BMEs to support organoid generation being attributed to their high abundance of laminins [[83], [84], [85], [86]]. Our analyzes revealed that the most abundant laminin α-, β-, and γ-chain-encoding proteins in the lung ECM and dECM were LAMA3, LAMB3, and LAMC2, indicating that the most common laminin trimer present within these samples was laminin-332, with other trimers, such as laminin-211 and -521 also being possible (Table S7) [86,87]. Regarding the lung, laminin-332 is a major component of the bronchioalveolar basement membrane, and can promote lung epithelial cell attachment, survival, proliferation and migration through interactions with cell intergrins α3β1 or α6β4 [88,89]. In the TME, laminin-332 can induce the formation of cancer cell lamellipodia, and highly promotes cancer cell adhesion and migration [[90], [91], [92]]. Laminin-332 also increases cancer cell EMT and migration in lung cancer cells [93]. Therefore, the high abundance of LAMA3, LAMB3, and LAMC2 within our ECM and dECM samples indicated they would support cell adhesion, migration and proliferation *in vitro*. In contrast, the most abundant laminin proteins found in the BME was LAMA1, LAMB1, and LAMC1, corresponding to laminin chains -α1, -β1, and -γ1, which comprise the laminin trimer laminin-111. Our results were consistent with previously reported proteomic analyzes of the commercially available BME, Matrigel [94,95]. Laminin-111 contains the peptides IKVAV and C16, which promote tumor growth and angiogenesis through interactions with integrin and integrin receptors, and AG73, that promotes tumor growth and metastasis via interaction with syndecan receptors [96]. Previous reports have noted that despite the functional utility of laminin-111 for *in vitro* applications, it is most commonly found in foetal tissues, being most functionally active during embryogenesis, and is rarely found in adult tissues [97,98].

Proteoglycans (PGs) were also highly abundant in the lung ECM and dECM samples, with the most abundant being heparan sulfate proteoglycan 2 (HSPG2), biglycan, decorin, lumican, agrin, and osteogylcin (Fig. 2, Table S6). Akin to the previously discussed collagens and laminins, HSPG2 and agrin are predominantly localized within the basement membrane, and are highly expressed in vasculature, conducting proangiogenic functions and maintaining basement membrane structural integrity [99,100]. Meanwhile, biglycan, decorin, lumican and osteoglycan regulate collagen fibrillogenesis through collagen fibril spatial organization [101]. The most abundant PG in the BME was HSPG2, accounting for over 96 % of the PGs.

In addition to the core matrisome, a variety of matrisome-affiliated proteins that perform specific ECM interactions were observed in the lung ECM and dECM (Fig. S4). Of these matrisome-affiliated proteins, the growth factors are arguably the most influential on the ECM and residing cells, due to their potency in directing cell behaviour and pivotal roles in various homeostatic and disease states. For example, TGF-β1 regulates upstream ECM processes such as the transcription of ECM proteins, such as laminin-332, and downstream processes such as EMT in cancer cells [[91], [92], [93], [94]]. Furthermore, there was a significant disparity in growth factors when comparing the lung matrices to the BME (Fig. S4C).

Overall, our analyzes revealed that the matrisomes of the lung ECM and dECM were highly constituted of proteins localized within the basement membrane, as shown by the high proportion and abundance of proteins such as collagen VI, HSPG2, agrin, and laminin. These proteins share similar roles in ECM assembly and maintenance, as well as cell anchoring, adhesion, proliferation, and migration. Our results indicated that key ECM components within the lung ECM were retained post-decellularization, therefore suggesting that the resulting dECM would maintain characteristics of the native lung tissue that are amenable for future cell culture and *in vitro* investigations. Additionally, although BME-based matrices have been shown to support pro-organoid and pro-tumoral behaviour *in vitro*, our analyzes show that the BME matrisome is non-representative of native lung tissue and could therefore cause cells to behave in a non-tissue representative manner compared to the lung dECM [95].

### 2.3 Methacrylation of lung dECM and LungMA hydrogel preparation

**Fig. 3:**
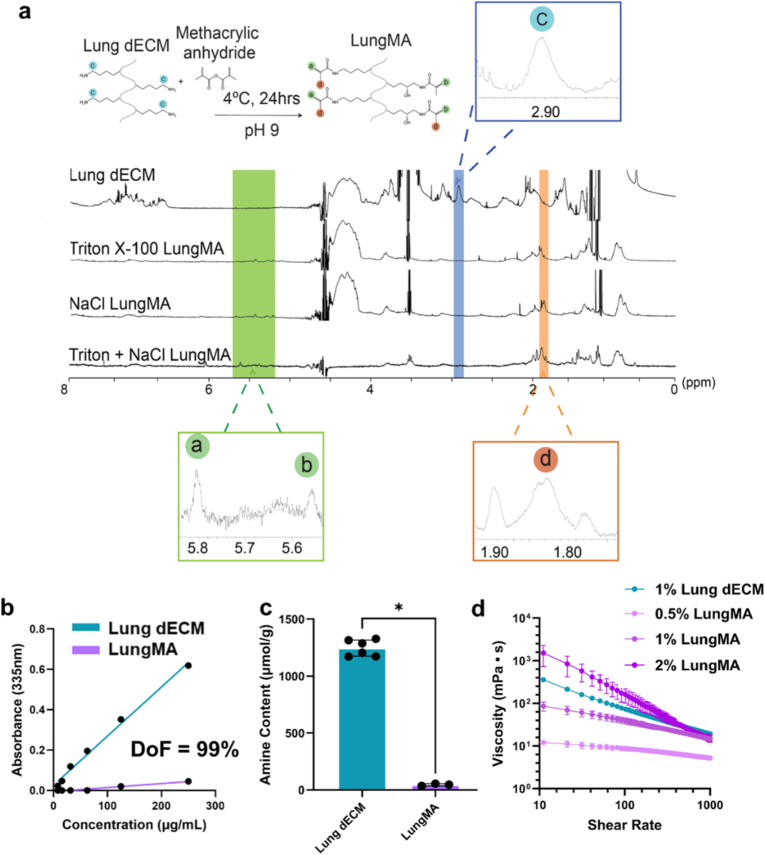
**Functionalization of lung dECM to prepare LungMA solutions. (A)** Proton nuclear magnetic resonance (^1^H NMR) spectra of lung dECM and LungMA. New peaks in LungMA groups at 5.8 ppm (a), 5.6 ppm (b) and 1.85 ppm (d) are indicative of newly grafted methacryloyl groups. Reduction in peak (c) at 2.99 ppm indicates loss of lysine-based amines due to functionalization. **(B)** Degree of functionalization (DoF) of LungMA, quantified via 2,4,6-Trinitrobenzene Sulfonic Acid (TNBS) assay. N = 1. n = 3–6. **(C)** Amine content of lung dECM and LungMA determined via TNBS assay. N = 1. n = 3–6. Mann Whitney test was used to analyze data. **(D)** Flow profiles of LungMA precursor solutions determined through shear rate sweeps demonstrate shear-thinning of properties of 2 % (wt/v) and 1 % (wt/v) LungMA solutions, and Newtonian behavior 0.5 % (wt/v) LungMA. N = 1. n = 3. All data is represented as mean ± SD. ∗*p* < 0.1.

Following decellularization, the lung tissue ECM and various dECMs were digested using pepsin for 96 h at room temperature, resulting in solubilized dECMs. As seen in Figure, the soluble protein concentration of lung ECM and dECM solutions increased over digestion time, with final concentrations ranging from 8 to 10 mg/mL (Fig. 3B). This concentration was higher than SLES-treated dECM (6 mg/mL, Fig. S5), suggesting that SLES decellularization either reduced soluble protein content or impaired solubilization. Our results reflect higher protein concentration compared to those previously reported by Pouliot et al., where digestion of lung dECM at 10 mg/mL yielded 5 mg/mL of total ECM protein after 72 h of digestion [25]. They also found that increasing digestion time is correlated with increasing dimeric and monomeric collagen I, and decreasing collagen I trimers, which may impact cell behaviour through biological binding, or changes in matrix stiffness, as dECMs digested for 48 and 72 h possessed a significantly lower storage modulus compared to dECMs digested for 4 and 12 h. These considerations call for potential future optimisation of digestion parameters in regard to collagen trimer cleavage, and how the presence or lack of collagen trimers effects LungMA preparation and use in cell culture.

Once digested, the dECM solutions were methacrylated via reaction with methacrylic anhydride at 4 °C for 24 h at pH 9, producing LungMA (Fig. 3A). To determine the molecular composition of the lung dECMs post-functionalization, proton nuclear magnetic resonance (^1^H NMR) was conducted on functionalized and unfunctionalized dECM solutions. As seen in Fig. 3A, all LungMA spectra exhibited a reduction in lysine (peak c, 2.90 ppm) compared to the dECM, representing the grafting of methacryloyl groups to free amines of the lung dECM [79,80]. Additionally, new peaks related to methacrylation were observed in the LungMA spectra at 5.6 ppm (peak b), 5.8 ppm (peak a) and 1.8 ppm (peak d), corresponding to the acrylic protons (peak a and peak b) and methyl protons (peak d) of the grafted functional groups [[102], [103], [104], [105], [106]]. We further quantified the degree of amine functionalization within the dECM and LungMA via 2,4,6-Trinitrobenzene Sulfonic Acid (TNBS) assay (Fig. 3C). LungMA possessed a high degree of functionalization (DoF), with 99 % functionalization of lung dECM lysine and hydroxy-lysine groups, and the total concentration of free amines in the lung dECM being reduced from 1246 ± 70 μmol/g to 45 ± 10 μmol/g in the LungMA (Fig. 3C). Following preparation of the LungMA hydrogel precursor solutions, the flow properties of the dECM and LungMA solutions were determined through shear rate sweeps. As seen in Fig. 3D, the viscosity of the LungMA solutions increased with material concentration, as did shear-thinning behavior [107,108].

**Fig. 4:**
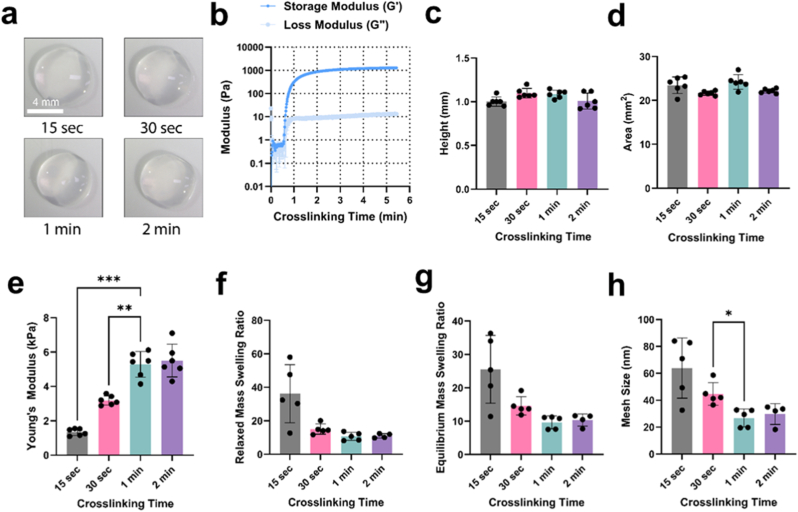
**Physicochemical properties of LungMA hydrogels. (A)** Stereoscopic images of LungMA hydrogels crosslinked with (lithium phenyl (2,4,6-trimethylbenzoyl) phosphinate) LAP under visible light (405 nm). Scale bar = 4 mm, magnification = 0.67×. **(B)** Time sweeps of LungMA hydrogels show increasing storage modulus (*G′*) with increasing crosslinking time. n = 3. Height **(C)**, area **(D)**, and Young's modulus **(E)** of 1 % (w/v) LungMA hydrogels crosslinked for 15 s, 30 s, 1 min, and 2 min show that hydrogel area and height is not significantly impacted by increased crosslinking time and Young's modulus. Hydrogel height and Young's modulus were determined via compression testing. Hydrogel area was determined through ImageJ analysis of stereoscopic images of hydrogels pre-compression. N = 1. n = 3. Relaxed mass swelling ratio **(F**), equilibrium mass swelling ratio **(G),** and mesh size **(H)** were determined via swelling and lyophilization of hydrogels**.** N = 1. n = 4–5. Non-parametric one-way ANOVA was used for intergroup comparisons. All data is represented as mean ± SD. ∗*p* < 0.1, ∗∗*p* < 0.01, ∗∗∗*p* < 0.001.

After characterizing the flow properties of LungMA precursor solutions, we assessed the physical properties of 1 % (w/v) LungMA hydrogels. Through photorheology, we observed rapid crosslinking, with hydrogels exhibiting storage modulus (*G′*) greater than loss modulus (*G″*) at the point of crosslinking, with storage modulus gradually increasing over crosslinking time, resulting in a final *G′* of 1302 ± 56 Pa at 5 min of crosslinking (Fig. 4B). The fast crosslinking of LungMA hydrogels here may have been caused by the material's high DoF (99 %, Fig. 3B), as increasing DoF in methacrylated hydrogels has previously been correlated with crosslinking time and Young's modulus [109,110]. The Young's modulus of LungMA hydrogels was able to be tuned by altering crosslinking time to match healthy (1–2 kPa, 15 s crosslinking time) and fibrotic (>4.5 kPa, >2 min crosslinking time) lung tissue stiffnesses (Fig. 4E) [26,35,111,112]. Increasing the Young's modulus appeared to make no significant differences in LungMA hydrogel macroscopic appearance (Fig. 4A), heights (Fig. 4C) or areas (Fig. 4D). These results are similar to what we observed in GelMA hydrogels, where increasing crosslinking time did not significantly impact hydrogel area, height, or macroscopic appearance (Fig. S7). Variation of photocrosslinking time enabled reproducible tuning of GelMA hydrogels' Young's modulus, with 1 min of crosslinking producing approximately 1 kPa and 8 min producing approximately 4 kPa (Fig. S7). GelMA precursor solutions were maintained at 37 °C prior to crosslinking and hydrogels were cultured at 37 °C thereafter, ensuring that stiffness modulation resulted solely from photocrosslinking rather than thermal gelation, which yields substantially lower modulus hydrogels [113]. Similar limitations apply to non-functionalized dECM hydrogels, which rely on thermal self-assembly and typically form soft matrices in the sub-kilopascal range (Table S12.2). Photocrosslinking therefore enables access to physiologically relevant stiffnesses that are difficult for thermal gelation alone to achieve, and allows LungMA and GelMA hydrogels to be precisely stiffness-matched for downstream studies.

Increasing LungMA hydrogel stiffness resulted in decreased mesh size, as seen in Fig. 4H, where mesh sizes varied from 63.96 ± 22.38 nm in LungMA hydrogels crosslinked for 15 s, to ∼30 nm in LungMA hydrogels crosslinked for 1 and 2 min. These results are similar to previous reports of GelMA-based hydrogels, where porcine-derived GelMA crosslinked for 2 min exhibited a mesh size between 53 and 59 nm, similar to that obtained in soft (15 s crosslinked) LungMA hydrogels [114]. Previous reports utilizing methacrylated hydrogels have also found that increasing crosslinking time is related to decreased mesh size, therefore suggesting that matrix stiffness is a result of decreased matrix mesh size, and that this characteristic may impact molecular diffusion within the hydrogel [[114], [115], [116], [117], [118], [119], [120]].

### 2.4 NSCLC cell morphology and response to chemotherapeutic treatment is dependent on matrix type and stiffness

After characterizing the physical properties of LungMA hydrogels, we optimized A549 NSCLC seeding densities to assess cell growth in LungMA and GelMA hydrogels with matched stiffness (1 kPa, Fig. S8) and found that cells remained viable over the course of culture independent of cell seeding density (Fig. S8B)We then aimed to determine the effect of both matrix stiffness and matrix type on A549 cell growth and behavior, by utilizing soft (1 kPa) and stiff (4 kPa) LungMA and GelMA hydrogels, in addition to BME. Encapsulated cells were characterized over time for morphology, DNA content and metabolic activity. Regarding matrix type, we hypothesized that cells cultured in the BME would behave more similar those in soft LungMA hydrogels compared to the other photo-crosslinked matrices. This is due to both BME and lung dECM being comprised predominantly of basement membrane proteins, whereas GelMA's precursor, gelatin, is mostly consistent of collagen type I and III, and inherently lacks basement membrane proteins such as laminins, due to it being a collagen derivative [104,121]. Additionally, BME possesses low stiffness, with Young's modulus reportedly ranging from (0.1–1 kPa) [[122], [123], [124]].

**Fig. 5:**
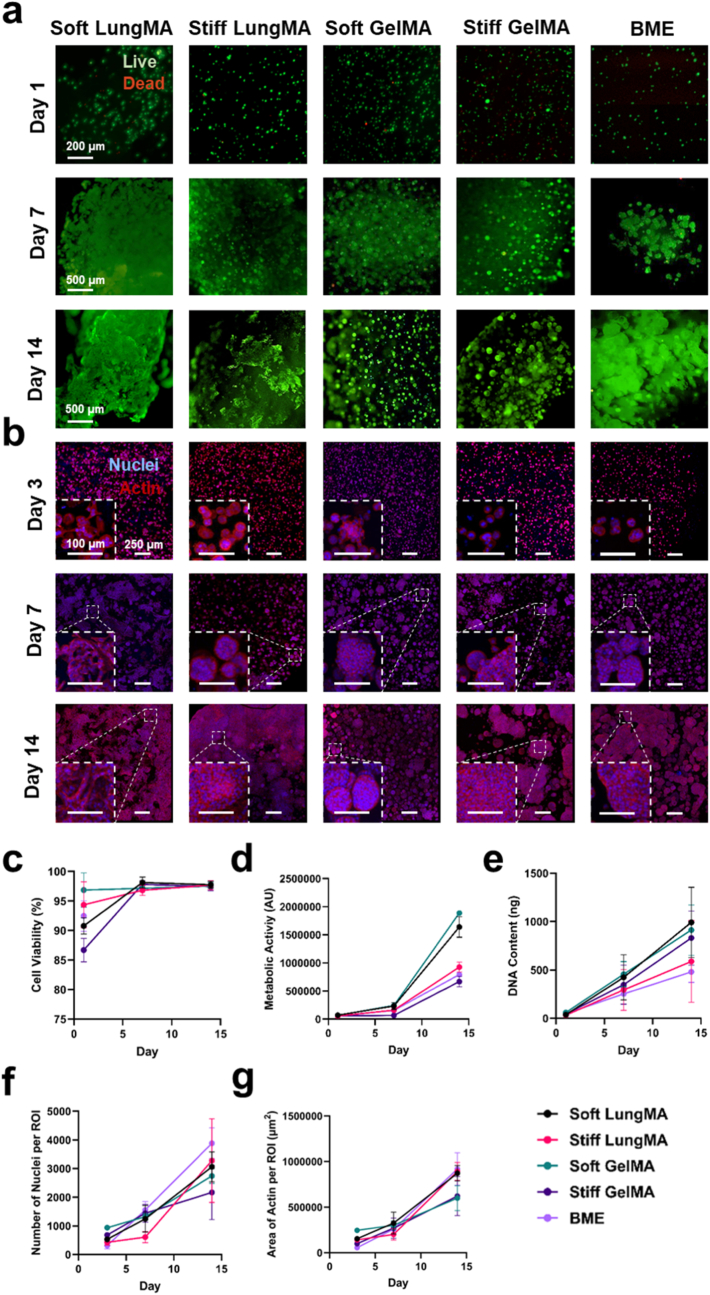
**NSCLC growth is dependent on matrix type and stiffness. (A)** Viability staining of encapsulated A549 cells using fluorescein diacetate (FDA, green, live) and propidium iodide (PI, red, dead). Day 1 scale = 200 μm, day 7 and 14 scales = 500 200 μm. **(B)** Representative fluorescent images of A549-laden cultures fixed on days 3, 7, and 14 and stained with DAPI (blue, nuclei) and phalloidin (red, actin) to observe cell morphology and distribution over time. Scale = 250 μm, inset scale = 100 μm. **(C)** Cell viability of A549s within matrices measured through live-dead quantification of FDA or PI stained live (green) and dead (red) cells over time via ImageJ analysis. N = 1. n = 3. **(D)** Metabolic activity of A549 cells within matrices was determined by PrestoBlue metabolic assay. N = 1. n = 6. **(E)** DNA content of A549 cells within matrices was determined by PicoGreen™ DNA quantification. N = 1. n = 6. **(F)** Number of nuclei per ROI was determined via Image J analysis of DAPI-stained cells within ROIs captured using an Evident FV4000 confocal microscope at 10× magnification. N = 2. n = 4–8 ROIs were captured per condition per timepoint. **(G)** Area of actin per ROI (μm^2^) was determined through ImageJ analysis of phalloidin-stained cell actin fibers. N = 2. n = 4–8. All data is represented as mean ± SD.

All matrices supported high A549 viability (>85 %) and proliferation over time, with metabolic activity, DNA content, and morphology being dependent on matrix type and stiffness (Fig. 5). Soft LungMA, and soft GelMA exhibited the highest metabolic activity at day 14 (Fig. 5D), while softstiff LungMA, and soft GelMA presented with higher DNA content than stiff GelMA and BME (Fig. 5E). These findings suggest that the 1 kPa microenvironments provided by soft LungMA and soft GelMA may enhance cell proliferation and metabolic activity.

Cell morphology was matrix-dependent over time, with differences most pronounced during late-stage culture (day 7- day 14) (Fig. 5, Figure S10.1 and S10.2). Notably, all matrices, with the exception of soft LungMA, promoted spheroid formation by day 7 of culture, which can contribute towards chemoresistance [[125], [126], [127], [128], [129], [130], [131]]. Quantification of cell nuclei via ImageJ analysis revealed a similar number of nuclei when comparing all matrices (Fig. 5F), however, quantification of actin staining revealed a higher amount of actin present per ROI in soft LungMA, stiff LungMA, and BME compared to soft and stiff GelMA conditions on day 14 of culture (Fig. 5G), suggesting increased cell spreading within the laminin-containing conditions. Soft LungMA hydrogels appeared to support a more elongated and migratory phenotype, accompanied by a significantly reduced e-cadherin signal (Fig. S12). Similar morphological features have been associated with early EMT when cells are exposed to altered matrix mechanics or TGFβ-1 cues, a growth factor which is present within the lung dECM (Fig. S4), and is characterized by cell migration, junction loss, and actin reorganization [103]. The decrease in e-cadherin may also be related to cell-ECM interaction with laminin-332, which promotes lamellipodia formation and migration [[87], [88], [89], [90], [91], [92], [93], [94]]. However, these observations remain speculative within the context of our study, and would need to be validated through in-depth characterization of EMT markers such as CDH1, VIM, and ZEB1 through RT-qPCR or Western blotting, together with cytokine profiling, including TGFβ-1 through ELISA [102,103].

Whilst investigating the effect of matrix stiffness on cell behaviour, we also investigated the effect of cell culture on matrix physical properties, such as Young's modulus, with the overarching goal being to determine whether cell-induced matrix degradation would affect gel properties. Interestingly, we found that culture of A549s for 7 days inside the respective hydrogel conditions resulted in a decrease in Young's modulus for all matrices when compared to their cell-free counterparts, while maintaining hydrogel height and area (Fig. S9). As seen in Fig. S9A, the Young's modulus of soft LungMA was reduced to approximately 0.5 kPa, stiff LungMA to 2.5 kPa, soft GelMA to 0.5 kPa, and stiff GelMA to 1.8 kPa. Although the Young's modulus of cell-free BME was not evaluated in this study, as previously mentioned, cell-free BME formulations have been reported as having Young's modulus ranging from roughly 0.1–1 kPa [[122], [123], [124]]. Here, BME cultured with A549s possessed an average Young's modulus of 0.15 kPa, suggesting that cell culture may have reduced the matrix Young's modulus. Observed reductions in Young's modulus may be due to matrix metalloproteinase (MMP)-2 and MMP-9-based enzymatic digestion, which targets type IV and V collagen, fibronectin, laminin, and elastin, thereby resulting in matrix remodelling [[131], [132], [133]]. Previously, Narasimhan et al. found that increasing the degradation potential of collagen matrices through the introduction of macromolecular crowding agents such as poly(ethylene glycol) resulted in decreased Young's modulus in HFF, and C212, and MCF10A-laden matrices compared to non-crowded samples [134]. They found that cells were able to sense MMP-based matrix degradation, and that in response, cells exhibited more elongated morphologies compared to their non-crowded counterparts. Therefore, differences in MMP-mediated matrix degradation may have also contributed towards cell morphology difference in A549s, such as tight spheroid formation in stiff LungMA, compared to more elongated cells in soft LungMA (Fig. 5B). Although it is not within the scope of this study, future studies should investigate the secretion of MMPs within the respective matrices, to determine whether such processes are matrix-dependent. Overall, these results highlight the role of matrix composition and stiffness in regulating lung cancer cell morphology.

Once determining the effect of matrix type on A549 NSCLC growth, we sought to determine whether A549 response to chemotherapy would be dependent on the culture matrix. Our selected chemotherapeutic agents were doxorubicin and cisplatin. These chemotherapeutic agents were chosen due to their difference in mechanisms of action, and frequency of clinical use for treatment of NSCLC. Doxorubicin promotes cancer cytoxicity through DNA intercalation, which prevents replication and transcription, in addition to generation of reactive oxygen species, which induces apoptosis, however, it is not a frontline therapeutic for NSCLC treatment, as NSCLC cells typically express drug efflux pumps such as P-glycoprotein, which inhibits intracellular accumulation of doxorubicin [[135], [136], [137], [138]]. In contrast, cisplatin is a frontline chemotherapeutic agent used to treat all stages of NSCLC, that functions through the formation of DNA crosslinks, and is not a substrate for P-glycoprotein, meaning cisplatin is commonly retained within cells over time, conferring its use for NSCLC treatment [[139], [140], [141], [142], [143]].

Chemotherapeutic agents were administered on day 3 and day 5 of culture, with doses ranging from 1 × 10^−4^ μM–100 μM for doxorubicin, and 1 × 10^−3^ μM–1000 μM for cisplatin. Response to treatment was measured on day 7 of culture, through assessment of metabolic activity normalized to vehicle controls. The IC_50_ for each group was generated via [inhibitor] vs. response variable slope (four parameters) non-linear regression. We hypothesized that A549 response to treatment would be dependent on matrix type, and that cells cultured in soft LungMA hydrogels would be less chemoresistant compared to cells cultured in other matrices. This was based on the formation of chemoresistant cell morphologies, as untreated A549s cultured in the BME, GelMA hydrogels and stiff LungMA hydrogels formed cohesive spheroids (Fig. 5B), which have previously been associated with increased chemoresistance, while A549s in soft LungMA aggregated into multicellular sheets and irregular spheroids [[140], [141], [142], [143], [144], [145], [146], [147]].

**Fig. 6:**
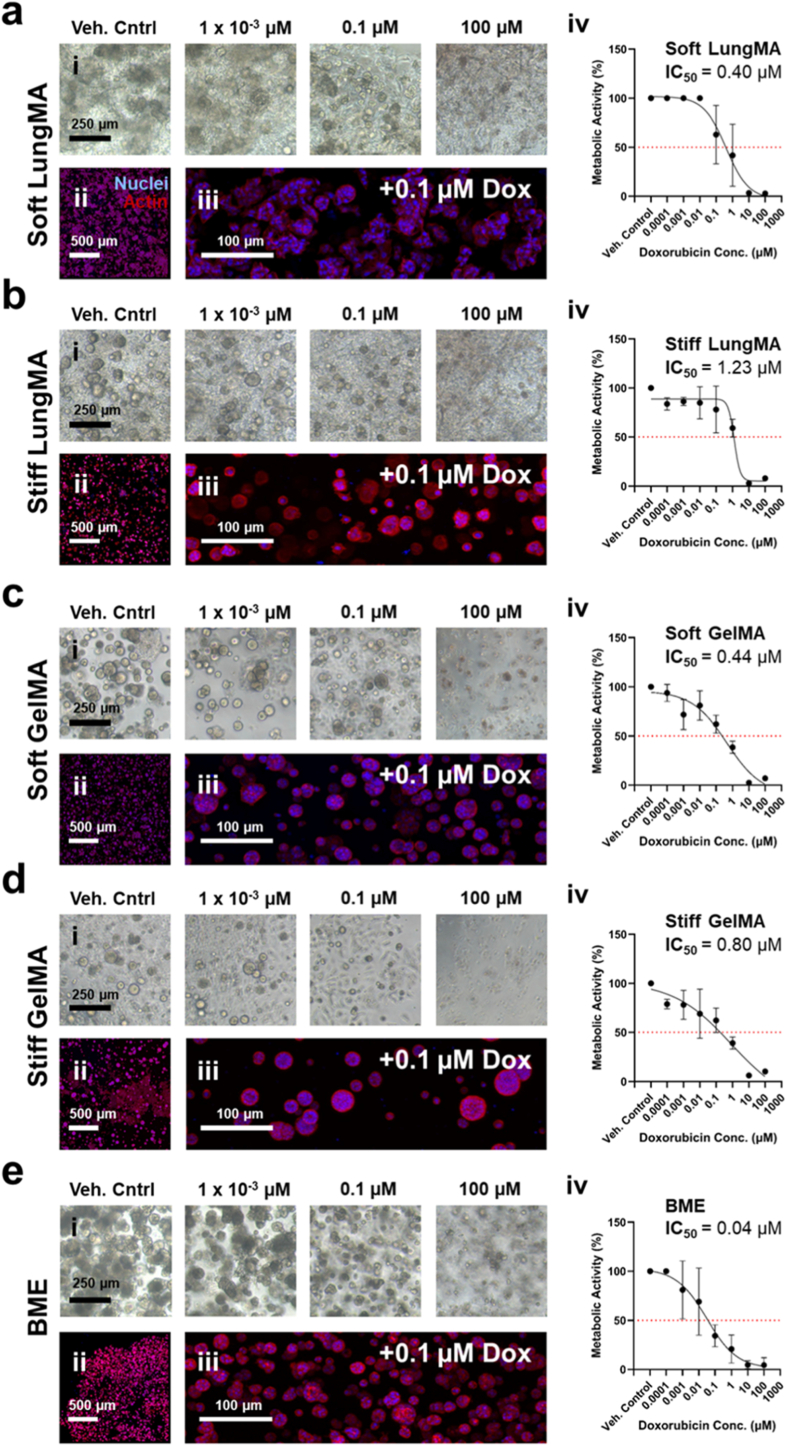
**Matrix-dependent NSCLC response to doxorubicin treatment**. A549s were encapsulated into **(A)** soft LungMA, **(B)** stiff LungMA, **(C)** soft GelMA, **(D)** stiff GelMA, or **(E)** BME hydrogels and treated from day 3 to day 7 of culture with dimethyl sulfoxide (DMSO) (vehicle control) or 7 respective concentrations of doxorubicin in DMSO ranging from 1 × 10^−4^ μM–100 μM. **(i)** Representative brightfield images of doxorubicin-treated cell cultures at endpoint of treatment. Scale bar = 250 μm. **(ii)** Low magnification view of cultures treated with 0.1 μM doxorubicin. Scale bar = 500 μm. **(iii)** High magnification view of cultures treated with 0.1 μM doxorubicin. Scale bar = 100 μm. **(iv)** IC_50_ curves of A549s within 3D matrices treated with doxorubicin. Cell metabolic activity was measured by PrestoBlue assay and normalized to day 7 vehicle control. IC_50_ values were generated via [inhibitor] vs. response variable slope (four parameters) non-linear regression. N = 1. n = 6. All data is represented as mean ± SD.

As seen in Fig. 6, stiff matrices promoted the highest chemoresistance to doxorubicin, with stiff LungMA promoting an IC_50_ of 1.23 μM, and stiff GelMA promoting an IC_50_ if 0.80 μM. Meanwhile, soft LungMA, soft GelMA, and BME, which possess stiffnesses within the range of healthy lung tissue, exhibited IC_50_ values of 0.40, 0.44, and 0.04 μM, respectively. These results suggest that chemoresistance was affected predominantly by matrix stiffness regardless of matrix type when A549s were treated with doxorubicin.

**Fig. 7:**
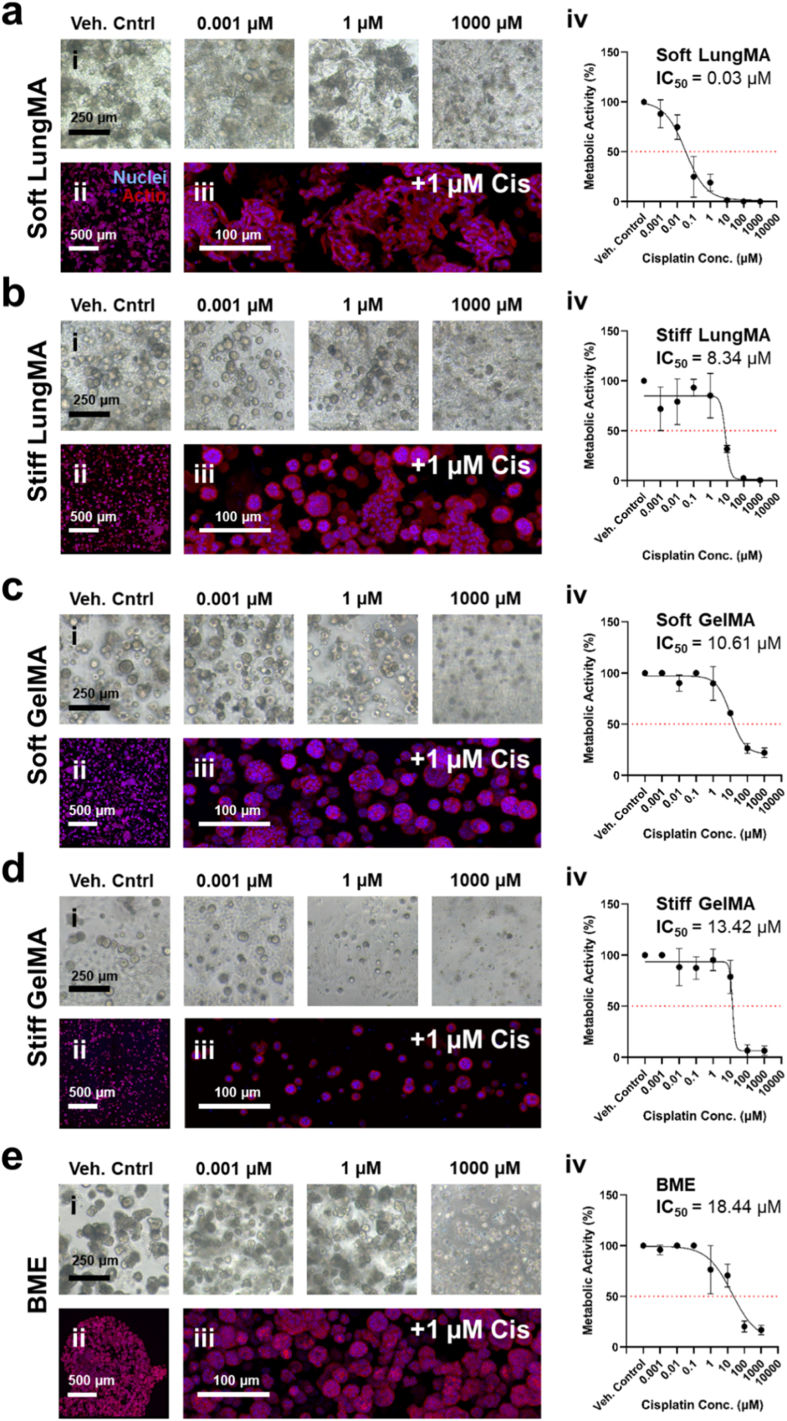
**Matrix-dependent NSCLC response to cisplatin treatment**. A549s were encapsulated into **(A)** soft LungMA, **(B)** stiff LungMA, **(C)** soft GelMA, **(D)** stiff GelMA, or **(E)** BME hydrogels and treated from day 3 to day 7 of culture with ultrapure water (vehicle control) or 7 respective concentrations of cisplatin in ultrapure water ranging from 1 × 10^−3^ μM–1000 μM. **(i)** Representative brightfield images of cisplatin-treated cell cultures at endpoint of treatment. Scale bar = 250 μm. **(ii)** Low magnification view of cultures treated with 1 μM cisplatin. Scale bar = 500 μm. **(iii)** High magnification view of cultures treated with 1 μM cisplatin. Scale bar = 100 μm. **(iv)** IC_50_ curves of A549s within 3D matrices treated with cisplatin. Cell metabolic activity was measured by PrestoBlue assay and normalized to day 7 vehicle control. IC_50_ values were generated via [inhibitor] vs. response variable slope (four parameters) non-linear regression. N = 1. n = 6. All data is represented as mean ± SD.

In contrast, a substantial difference in response to treatment profiles was observed during treatment with cisplatin, where all matrices, with the exception of soft LungMA, exhibited IC_50_ values ranging between 8 and 18 μM cisplatin (Fig. 7). When treated with cisplatin, soft LungMA promoted an IC_50_ of 0.03 μM.

When comparing the effect of drug treatment on cell morphology, chemoresistance appeared to be related to the area of actin per ROI of treated samples (normalized to day 7 untreated samples), where the greater resistance to treatment was mostly consistent with a higher normalized area of actin per ROI (Fig. S12). For example, soft LungMA exhibited greater chemoresistance to doxorubicin than to cisplatin, which is reflected by the normalized area of actin per ROI being higher in doxorubicin treated samples compare to those treated with cisplatin. Meanwhile cells cultured in stiff LungMA and soft GelMA possessed higher chemoresistance to cisplatin than to doxorubicin, which was also reflected by their normalized area of actin per ROI in treated samples. However, this trend was not evident in stiff GelMA and BME therefore, the relationship between chemoresistance determined by metabolic activity, and area of actin, is not apparent for all matrices.

These results highlight that matrix stiffness-induced chemoresistance may be dependent on the mechanisms of action of the chemotherapeutic of interest, and how such mechanisms influence cell-ECM interactions, which are themselves influenced by matrix-specific microenvironment stiffness and protein compositions [[144], [145], [146], [147], [148], [149], [150], [151], [152], [153], [154]]. For example, increased ECM stiffness can result in the activation of focal adhesion kinases (FAKs), which activate mitogen-activated protein kinase (MAPK) pathways that can promote cell proliferation and survival [[129], [130], [131], [132]].

In terms of ECM composition, the concentration and type of binding motifs present may influence the ability for cells to migrate and adhere to ECM, thereby promoting differential responses to treatment. In this study, the lung dECM used for preparation of the LungMA hydrogels possessed laminin-332 (Table S7), which contains laminin-specific binding motifs YIGSR and laminin G-like (LG) domains, that promote cell spreading and migration, in addition to arginylglycylaspartic acid (RGD), which generally promotes stronger cell adhesion compared to its laminin-based counterparts, and is more sensitive to ECM stiffness [[155], [156], [157], [158], [159], [160], [161], [162], [163], [164]]. Meanwhile, GelMA hydrogels are derived from gelatin, and therefore do not possess laminin-based binding motifs, instead relying primarily on RGD motifs for cell binding [[165], [166], [167]]. The presence or lack of such motifs, in addition to their distribution and concentration, may influence the activation of chemoresistant pathways such as FAKs, and thus, chemoresistance.

The influence of matrix composition on drug-cell-ECM interactions can be seen through comparison of stiffness-matched LungMA and GelMA hydrogels, where similar stiffness-based response to therapy trends were observed in LungMA and GelMA (increased stiffness promotes increased chemoresistance) during treatment with doxorubicin (Fig. 6), but such effects were not as substantial during treatment with cisplatin (Fig. 7).

Here, soft LungMA may have been more susceptible to cisplatin than doxorubicin treatment for various reasons, such as cisplatin's inhibition of A549 integrin-ECM binding. [143] Integrins facilitate cellular adhesion to ECM binding motifs such as those listed previously, allowing spheroid formation. As seen in Fig. 5, Fig. 6, Fig. 7, A549s cultured in soft LungMA appeared to form non-spheroidal multicellular aggregates without treatment, reflective of laminin-based cell integrin-ECM adhesion to YIGSR [[159], [160], [161], [162], [163], [164]]. Disrupting integrin-ECM binding at early stages of culture, such as the time of first cisplatin dose (day 3) may have prevented cells from forming spheroids over time, therefore reducing their potential chemoresistance. In contrast, A549s cultured in stiff LungMA, soft and stiff GelMA, and BME, appeared to form more spheroid-like morphologies prior to the treatment period (Fig. 5B), and after (Fig. 6B-E), potentially due to ECM stiffness-based mechanotransduction and upregulation of RGD-based binding in the photocrosslinkable matrices [[149], [150], [151], [152], [153], [154],[165], [166], [167]], and IKVAV-mediated polarity signalling in BME, ultimately increasing cisplatin chemoresistance [[168], [169], [170], [171]].

Integrin-mediated spheroid formation may have also played a role in the similar chemoresistance profiles and morphologies observed in doxorubicin treated A549 cultures in soft LungMA and stiff LungMA (Fig. 6A and B), and soft and stiff GelMA (Fig. 6C and D), where cell clusters appeared more spheroid-like compared to those seen in soft LungMA treated with cisplatin. Unlike cisplatin, doxorubicin doesn't directly target cell integrin binding, instead, integrin expression has been linked with chemoresistance [[172], [173], [174]]. This may explain why A549s in soft LungMA treated with doxorubicin appeared to undergo adaptive spheroid formation in response to doxorubicin treatment, as seen in brightfield and fluorescent images of treated cultures (Fig. 7A), while such morphologies weren't as common during cisplatin treatment. In addition to integrin binding, many other ECM-cell-drug interactions could be attributed towards agent-specific chemoresistance, such as downregulation of MMPs. For example, previous works have demonstrated that A549 MMP-2 and MMP-9 expression was effectively supressed during treatment with doxorubicin, but relatively unaffected during cisplatin treatment [[175], [176], [177]].

Despite such interactions being of great interest, here they are merely speculative, and further investigations into ECM-cell-drug interactions should be conducted in the future, to validate such hypotheses. Although this work does not investigate the role of ECM-cell-drug interactions mechanistically, the difference in response-to-treatment exhibited by A549s regarding cell morphology and metabolic activity demonstrates the importance of matrix composition and stiffness on cell behaviour, and how these factors can promote or reduce chemosensitivity and response to treatment. Overall, with the exception of soft LungMA, it appeared that A549s were more susceptible to treatment with doxorubicin (Fig. 6) compared to cisplatin (Fig. 7), as demonstrated by the greater IC_50_ values observed in groups treated with cisplatin. The stiffness-dependent response to treatment of A549s cultured in either native lung-mimicking stiffness (1 kPa) or fibrotic lung-mimicking stiffness (4 kPa) LungMA hydrogels reflected results obtained in clinical studies, where fibrotic lung, such as in the case of idiopathic pulmonary fibrosis, is both a risk factor for the onset of lung cancer and majorly associated with poor lung cancer patient outcomes [[178], [179], [180], [181], [182], [183]]. No stiffness-dependent effects were seen in GelMA treated with the frontline chemotherapeutic cisplatin, despite its ability to be tuned to the same Young's modulus (Fig. 4, Fig. 7, and Fig. S7). Native dECM hydrogels were not included because their ability to reach physiologically relevant stiffnesses is variable, and dependent on varying material concentration (Table S12), making stiffness matching whilst maintaining consistent concentrations impossible, thereby confounding potential findings. The observed differences between stiffness-matched LungMA and GelMA therefore suggest that lung-specific matrix cues, rather than stiffness alone, drive enhanced chemosensitivity in LungMA. Our findings demonstrate the high potential and relevance of LungMA hydrogels for use in future lung-specific *in vitro* cell culture, cell-ECM interaction, and drug screening studies.

## 3 Conclusion and future directions

This study presents the development and *in vitro* application of a lung-specific hydrogel and its use for culture and treatment of NSCLC. This hydrogel utilizes highly controlled photocrosslinking for its formation. Our optimized dECM lacks native lung tissue cellular components, as demonstrated by a significant reduction in native tissue nuclei and DNA, while preserving native lung ECM proteins, as shown through proteomic analyzes. By employing photocrosslinking to generate LungMA hydrogels, we achieved precise control over matrix stiffness, allowing for the simulation of either healthy or fibrotic lung tissue depending on the crosslinking duration. We successfully demonstrated the utility of LungMA hydrogels for investigating lung cancer research questions *in vitro*. NSCLC cells encapsulated in these hydrogels exhibited growth characteristics and treatment responses that were dependent on the stiffness of the matrix. Notable differences in cell growth and response to treatment were observed in soft LungMA hydrogels compared to other matrices, displaying the critical role of tissue-specific cell-ECM interactions in influencing *in vitro* cell behavior and chemotherapeutic outcomes.

Although our current findings detail the potential LungMA hydrogels have for future applications in *in vitro* lung cancer cell culture studies, this study also possesses some experimental limitations. Regarding the synthesis of LungMA, ECM components could not be measured post-methacrylation, due to constraints regarding the ability for proteomic analyzes to detect functionalized ECM proteins as a result of chemical modification of amine groups, with less specific methods such as NMR being preferred [[104], [105], [106],^184^,^185^]. Therefore, there is some potential for loss of ECM proteins during functionalization, which should be investigated through techniques such as SDS-PAGE. Furthermore, we used 4 kPa matrices as fibrotic lung-representatives, however, lung fibrosis stiffness often exceeds this value (Table S12). Thus, future studies could include a LungMA condition with higher stiffness. Additionally, hydrogel mechanical properties were evaluated through rheology and compression testing (Fig. 4), however, through comparison of previously reported lung hydrogel stiffnesses, we found that stiffness was highly dependent on the technique being used (Table S12.2). Therefore, other techniques such as atomic force microscopy or microindentation could also be conducted, to further characterise the mechanical properties of the hydrogels at greater resolution.

Furthermore, only one cell line, A549, was used in assessing the effect of matrix type on cell growth. This cell line was chosen due to its previously reported high proliferation rate, ability to form spheroids in 3D matrices, and sensitivity to chemotherapy [[186], [187], [188]]. To overcome this limitation, future studies could include multiple lung cancer cell types to assess growth and response to treatment, such as HCC95, a human lung squamous cell carcinoma, or DMS273, a lung cancer cell line commonly used to study metastasis [[189], [190], [191], [192], [193], [194]], or patient-derived lung cancer cells. Although this study focused on the influence of matrix source and initial stiffness on NSCLC growth and therapeutic response, in addition to the effect NSCLC growth has on surrounding ECM mechanical properties, tumour driven ECM deposition is also an important contributor to disease progression. Lung adenocarcinoma cells have been shown to upregulate and secrete specific collagen isoforms that restructure the surrounding ECM and promote malignant behaviour. COL10A1 overexpression has been associated with ECM remodelling and tumour progression in lung adenocarcinoma [[195], [196], [197], [198]]. Quantifying *de novo* collagen synthesis within LungMA hydrogels would require extended culture periods and dedicated biochemical or molecular assays, such as hydroxyproline quantification, ELISA, or transcriptomic profiling, which lie beyond the scope of the present study. Nevertheless, the role of tumour derived collagen deposition represents a valuable direction for future investigation, particularly in relation to fibrosis associated cancer progression. Regarding mechanisms of action, the role of lung-specific cell-protein binding, such as cell integrin binding to laminin, and the effect of photocrosslinking on such mechanisms, was discussed, but not investigated mechanistically. Therefore, future studies could aim towards detecting specific cell-ECM binding patterns in soft and stiff matrices, to observe how binding characteristics are altered in stiffer environments, and their resulting influence on chemoresistance. Additionally, utilizing advanced fabrication techniques, such as micropatterning, in conjunction with direct ECM stiffness modulation using LungMA, would enable systematic investigation of how contact guidance, spatial confinement, and aggregate size interact with matrix mechanics to influence NSCLC growth and response to treatment [199]. This approach represents an important direction for future work. In conclusion, our findings highlight the significance of tissue specific matrix type and stiffness in cell culture and drug studies, emphasizing the high potential and utility of tissue-specific models for *in vitro* applications.

## 4 Experimental section/methods

### 4.1 Lung decellularization

Porcine lung tissue sourced from Australian pigs aged 5–6 months was collected from a butcher (QUT Tissue Use approval 6842) and stored at −80^0^C. Once thawed, a minimum of 3 pairs of lungs per batch (3 batches total) were segmented using a scalpel and homogenized using a stick blender. After homogenization, the lung tissue homogenates were then either stored at −80^0^C or decellularized immediately. Decellularization was performed using multiple respective protocols, each utilizing various decellularizing solutions and series (Table 1).

**Table 1:** Lung decellularization protocols.

Protocol	Description
NaCl	1 M NaCl (ChemSupply, Australia, lot# SA046-3 KG) for 6 days
Triton X-100	2 % (wt/v) Triton X-100 (Merck Millipore, USA, CAS# 9036-19-5) for 6 days
SLES	2 % (wt/v) SLES (All Chemical, Australia, lot# SLES7030) for 6 days
CHAPS	2 % (wt/v) CHAPS (Thermo Fisher Scientific, USA, lot# YB356412) for 6 days
Triton X-100 + NaCl	2 % (wt/v) Triton X-100 for 2 days, then 1 M NaCl for 4 days
SLES + NaCl	2 % (wt/v) SLES for 2 days, then 1 M NaCl for 4 days
CHAPS + NaCl	2 % (wt/v) CHAPS for 2 days, then 1 M NaCl for 4 days
2 M NaCl	2 M NaCl for 6 days
4 M NaCl	4 M NaCl for 6 days
6 M NaCl	6 M NaCl for 6 days

Porcine lung homogenates were decellularized at 0.5 g tissue/mL decellularizing solution in Bellco spinner flasks at 270 rpm, room temperature (RT, 25 °C) for 8 h. Then, the decellularizing solutions were exchanged for deionized (DI) water and washed for 30 min at RT. Once washed, the DI water was removed, and tissues were treated with respective decellularization solutions overnight. At timepoints (day 2, 4 and 6), lung tissue homogenates underwent 10 consecutive wash steps to remove residual contaminants, and dECM samples were collected. At decellularization endpoint (day 6), the remaining tissues were stored at −80 °C.

### 4.2 DNA quantification

Porcine lung tissue, dECM, or cell-laden hydrogels frozen at −80 °C were digested in 0.5 mg/mL Proteinase K in pH 7.1 phosphate-buffered EDTA (PBE) for 24 h at 65 °C. DNA quantification was performed using a Quant-iT™ PicoGreen Kit™ (Thermo Fisher Scientific) following the manufacturer's instructions and normalized to tissue and hydrogel wet weight.

### 4.3 Glycosaminoglycan (GAG) quantification

The sulphated glycosaminoglycan (sGAG) content of Proteinase K-digested lung tissue and dECM samples were determined using Dimethylmethylene Blue (DMMB) assay using DMMB solution (Sigma Aldrich) following the manufacturer's instructions. sGAGs were quantified using 525/595 nm absorbance ratio against a second-order polynomial standard curve and normalized to tissue wet weight.

### 4.4 Lung tissue and dECM paraffin embedding

Porcine lung tissue and dECM homogenates (weight 20–30 mg) were washed with 1 X PBS (Invitrogen, USA) for 15 min and fixed in 4 % (wt/v) paraformaldehyde (PFA) for 1 h. After fixing, PFA was removed, and tissue homogenates were washed thrice with PBS. Fixed tissues were then stored in 70 % (v/v) ethanol at 4 °C, or transferred directly into histology cassettes. The cassettes were placed in 80 % (wt/v) ethanol until processing using a Leica ASP300S overnight. Once processed, tissue homogenates were embedded in paraffin wax using a Leica HistoCore Arcadia H + C paraffin embedding station and cooled on a cold plate for 30 min. The embedded homogenates were then transferred to a previously prepared blank tissue microarray (TMA).

### 4.5 Preparation of tissue microarrays (TMAs)

TMAs molds were produced by 3D printing using a Formlabs 80 A flexible resin on a Formlabs 3B + SLA 3D printer. The TMA mold was inserted into a histological cassette, embedded with paraffin and cooled on a cold plate. After cooling, the embedded TMA mold was left at 25 °C for 1 h, then the mold was removed from the cassette, leaving the blank paraffin TMA on the cassette ready for sample insertion. Embedded tissue homogenates were then hole-punched and inserted into paraffin TMA using a biopsy punch. Any sample receptacles in the TME without sample were filled with additional paraffin to prevent slice tearing during microtomy. Once filled, the TMA was placed on a glass slide on plate heater set to 50 °C and heated for 1 min to even the TMA face.

### 4.6 Microtomy and histological staining

Tissue-filled TMAs were sectioned on a Leica 2245 semi-automatic microtome at slice thickness 4 μm. Tissue sections were placed in 37 °C water bath, then transferred to Polysine™ Adhesion Microscope Slides (Epredia, ref# J2800AMNZ) and dried in an oven for 2 h. Once dried, slides were stained using hematoxylin and eosin (H&E), Safranin-O (S-O) and Masson's trichrome (M-T) respectively using a Lecia XL Autostainer and imaged at 40 × magnification on a 3D Histech slide scanner microscope. For each condition, timepoint, and stain, section images were captured at 2 × (n = 1), 5 × (n = 1) 10 × (n = 1) and 40 × (n = 4) magnification on Caseviewer. Tissue nuclei were quantified using ImageJ software.

### 4.7 Proteomics

Proteomic lysis buffer was prepared using reagents as outlined in Table 2. Then, 5–15 mg of each porcine lung, lung dECM or growth factor reduced BME (Cultrex™) samples were sterile weighed into lo-bind tubes, and 500 μL of lysis buffer was added to each sample. Once lysis buffer was added, metallic processing beads were added to samples in lo-bind tubes. The bead-laden tubes were then added to a bead homogenizer and homogenized at speed 3 for 3 min or until tissue was homogenized. The homogenized samples were then submitted to the Translational Research Institute (TRI) proteomics core facility for assessment using an UltiMate™ NCS-3500RS Liquid Chromatography Pump with Capillary Flow Meter and Column Compartment (Thermo Scientific™). Matrisome proteins in generated datasets were then detected and identified using the Matrisome Project database (https://hynes-lab.mit.edu/resources/matrisome/fasta_files).

**Table 2:** Proteomics lysis buffer preparation.

Reagent	Final Concentration	% in Final Solution
Tris (pH 8.5)	100 mM	10
Sodium deoxycholate (SDC)	1 % (wt/v)	10
TCEP	10 mM	2
2-CAA	40 mM	10
Ultrapure water	N/A	68

### 4.8 Pepsin digestion

Lung tissue and dECM homogenates were solubilized through digestion of homogenates at 10 mg/mL with 0.5 mg/mL Pepsin (Sigma, USA, lot# P6887-54) in 0.01 M HCl at RT for 92 h in spinner flasks. Soluble protein concentration was measured at A_280nm_ using a NanoDrop One^C^ Microvolume UV–Vis Spectrophotometer.

### 4.9 Lung dECM methacrylation

Methacrylated lung dECM (LungMA) was prepared based on previously reported methods of gelatin methacrylation, with minor alterations [78,79,200]. At 4 °C, dECM solutions were added to stir flasks and the pH was adjusted to 9.0 ± 0.5 using 2 M NaOH. Then, methacrylic anhydride (Sigma-Aldrich, Germany) was added to the dECM solution at a ratio of 0.6 g MAAH/g sdECM. The pH was adjusted to 9.0 and the reaction was stirred at 4 °C overnight. Following the reaction, dECM-MA solutions were added to 3.5 kDa molecular weight cut off SnakeSkin™ dialysis membranes (Thermofisher, USA, lot# YF359205) and dialyzed against 0.1 mM HCl with conductivity monitoring using a Combi Point Tester (Thermofisher, USA) to determine the dialysis endpoint. Once dialysis was completed, LungMA solutions were transferred to reaction tubes (Thermofisher) and frozen overnight at −80 °C. Then, frozen LungMA was lyophilized using a Biobase Vertical Freeze Dryer (BK-FD18PT) for 3 days. Post-lyophilization, LungMA was stored at −80 °C until further use.

### 4.10 TNBS assay

The amine content of solubilized lung dECM and LungMA was quantified using 2,4,6-trinitrobenzenesulfonate (TNBS) assay as previously described. In brief, pepsin digested lung tissue and dECM homogenates were dissolved in 0.1 M NaHCO3 buffer to prepare a dilution series ranging from 250 μg/mL to 0 μg/mL. Triplicates of 200 μL of both lung dECM and LungMA solutions were transferred to a 96-well plate, mixed with 100 μL of 0.01 % (wt/v) TNBS solution, and incubated for 2 h protected from light. The plate absorbance was measured at 335 nm.

### 4.11 ^1^H NMR

^1^H NMR was conducted as previously described [78,79,200]. In brief, lyophilized lung dECM and LungMA samples were dissolved in 90 % H_2_O/10 % D_2_O solution to a final concentration of 1 % (wt/v). A volume of 1 mL of each sample was added to respective NMR tubes and sample spectra were collected using a Bruker Avance 600 MHz (Bruker, Billerica, MA, USA) with water suppression protocol. The collected sample spectra were analyzed using Bruker TopSpin 4.0.6 software.

### 4.12 Lung dECM and LungMA rheology

The rheological properties of lung dECM and LungMA solutions, and LungMA hydrogels were determined using an Anton-Paar modular compact rheometer (MCR) 302 (Anton-Paar, Graz, Austria). To determine flow profiles of lung dECM and LungMA, the solutions were diluted to a final concentration of 1 % (wt/v) for lung dECM using 1 X PBS, and final concentrations of 2 % (wt/v), 1 % (wt/v) and 0.5 % (wt/v) using 1 X PBS for LungMA. A 25m cone plate (CP25) was fitted to Anton-Paar MCR 302, and once calibrated, 110 μL lung dECM or LungMA solution was added to rheometer stage. Shear-rate sweeps were conducted at 25 °C, with shear-rate range of 0.1–1000/s, at a constant frequency of 1 Hz. n = 4. To determine the storage (G’) and loss (G”) modulus of LungMA hydrogels over crosslinking time, 110 μL of LungMA solution was added to the rheometer stage, and time sweeps were conducted using a PP25 at a constant frequency of 1 Hz and constant strain of 1 %, for 1 h n = 4.

### 4.13 Photocrosslinking of LungMA hydrogels

Lyophilized LungMA was dissolved to a final concentration of 1 % (w/tv) in 1 X PBS containing 0.15 % (w/tv) Lithium phenyl-2,4,6-trimethylbenzoylphosphinate (LAP) photoinitiator and adjusted to pH 7 using 1 M NaOH to prepare complete LungMA precursor solution. 30 μL samples of complete LungMA precursor solutions were photocrosslinked by exposure to 405 nm light (approx. 12 mW/cm2) in a LunaCrosslinker™ (Gelomics) for 15 s, 30 s, 1 min, and 2 min, respectively, forming LungMA hydrogels. Complete GelMA hydrogel precursor solutions were comprised of warmed 5 % (wt/v) GelMA (porcine skin, Type A, 80 % degree of functionalization, lot# GPM25C35, Gelomics) and 0.15 % (wt/v) LAP dissolved in PBS. 30 μL samples of complete GelMA precursor were photocrosslinked by exposure to 405 nm light (approx. 12 mW/cm2) in a LunaCrosslinker™ (Gelomics) for 1 min, 2 min, 4 min, and 8 min, respectively, forming GelMA hydrogels.

### 4.14 Hydrogel compression testing

Hydrogels were swollen overnight in PBS at 37 °C. Following swelling, hydrogels were imaged using a Nikon SMZ25 stereomicroscope at 0.46× magnification, and surface area of the hydrogels was determined using ImageJ software. The hydrogels were then submerged in a 37 °C PBS-filled water bath and compressed in an unconfined configuration using an Instron 5848 Microtester (Instron®, Norwood MA, USA) and non-porous aluminium indenter, at a strain rate of 0.01 mm/s. The Young's moduli (*E*) of the hydrogels were determined as the slope of stress-strain curves at 10–15 % strain.

### 4.15 Hydrogel swelling

The swelling properties of the hydrogels were determined as previously described for gelatin-based hydrogels [[115], [116], [117], [118]]. The hydrogels were weighed immediately post-crosslinking (*m*_*crosslinked*_), then swollen overnight in PBS at 37 °C. After swelling, hydrogels were re-weighed (*m*_*we*t_), then lyophilized, then weighed again (*m*_*lyophilized*_). The recorded weights of the hydrogels were used to calculate swelling ratios, alongside the mesh size (ξ), using the Flory-Rehner theory. The mesh size calculation is described in Equation (1), using the molecular weight of the repeating unit (M_r_), the amino acid bond length (*l*), Flory's characteristic ratio (C_n_) and the molecular weight between crosslinks (M_c_).

$$Theoretical\;mesh\;size\;\left(\xi \right)={v2s}^{-\frac{1}{3}}*l\;{\left(2\frac{M_{c}}{M_{r}}C_{n}\right)}^{\frac{1}{2}}$$

Here, parameters used were acquired based on previous protocols using similar ECM hydrogel formulations, and are listed in Table 3 [115].

**Table 3:** Parameters used to calculate theoretical mesh size.

Constant	Abbreviation	Value
Molecular weight of the repeating unit	Mr	91.19 g.mol−1
Amino acid bond length	l	4.25 Å
Flory's characteristic ratio	Cn	8.8785
Polymer density	ρp	1.35 g.cm−3
PBS solvent density	ρs	1.014 g.cm−3
Specific volume	*v*	0.7407 mL g−1
Molar volume of solvent	V1	18.01 mL mol−1
Flory's Chi parameter	X1	0.497
Average molecular weight before crosslinks	Mn	63 565.35 g.mol−1

### 4.16 A549 lung cancer cell culture

1 × 10^6^ A549 cells (ATCC) were seeded at P90 in T75 culture flasks and cultured using 10 mL complete A549 media (Dulbecco's modified eagle medium (DMEM) + 10 % FCS + 1 % pen/strep) in a tissue culture incubator at 37 °C. 5 % CO_2_, 95 % air. A549 cells were split at a ratio of 1:2 once 80 % confluent. The A549 cells were used under QUT Human Research Ethics approval 9140.

### 4.17 3D lung cancer cell culture

A549 cells were cultured as described previously, then trypsinized and seeded into new 15 mL Falcon tubes prior to centrifugation at 300*g* for 3 min. After centrifugation, the supernatant was removed, and cell pellets were resuspended in complete hydrogel precursor solutions at a concentration of 4 × 10^5^ cells/mL. Complete GelMA hydrogel precursor solutions were comprised of warmed 5 % (wt/v) GelMA (porcine skin, Type A, 80 % degree of functionalization) and 0.15 % (wt/v) LAP dissolved in PBS. Complete BME was comprised of 3 mg/mL growth factor reduced Cultrex® BME. Once resuspended, 30 μL aliquots of the cell-laden hydrogel precursor solutions were added to 24-well plates. Photocrosslinkable matrices crosslinked using 405 nm light (approx. 12 mW/cm^2^) in a LunaCrosslinker™ for 15 s (LungMA) or 1-min (GelMA) to achieve soft (∼1 kPa) matrices, and 1-min (LungMA) or 8 min (GelMA) to achieve stiff (∼4 kPa) matrices. BME matrix was crosslinked through incubation of hydrogel precursor solution at 37 °C, 5 % CO_2_, 95 % air in cell culture incubator for 30 min.

### 4.18 Live/dead viability assay

A549 cells were cultured as described previously, then trypsinized and seeded into new 15 mL Falcon tubes prior to centrifugation at 300*g* for 3 min. After centrifugation, the supernatant was removed, and cell pellets were resuspended in complete hydrogel precursor solutions at a concentration of 4 × 10^5^ cells/mL. Once resuspended, 30 μL aliquots of the cell-laden hydrogel precursor solutions were added to 24-well plates. Matrices were crosslinked as previously described. At timepoints (day 1 and day 7), hydrogels were washed with PBS thrice, then stained in viability staining solution comprised of 10 μg/mL fluorescein diacetate (FDA, Thermo Fisher Scientific) and 5 μg/mL propidium iodide (PI, Thermo Fisher Scientific) in PBS for 2 min. Once stained, cell-laden hydrogels were then washed twice with PBS and imaged using a Nikon® SMZ25 epifluorescent microscope. 3 ROIs per magnification (4× and 10 × ) per sample per timepoint were captured. Z-stacks of hydrogels were captured with 5 μm slice intervals at 10× magnification and 10 μm intervals at 4× magnification. Maximum intensity projections of subsequent z-stacks were obtained using ImageJ. n = 3.

### 4.19 Fluorescent imaging

Cell-laden hydrogels were fixed in 4 % (wt/v) PFA for 1 h, then washed and stored at 4 °C in PBS. The hydrogels were then incubated in blocking buffer (5 % (v/v) goat serum (Thermo Fisher Scientific) at 4 °C on plate shaker for 2 h. Samples were washed thrice with PBS, then e-cadherin primary antibody solution (ab40772, mouse monoclonal, Abcam) at a 1/200 dilution in washing buffer (20 % blocking buffer in PBS) was added to samples and incubated overnight at 4 °C on plate shaker. Then, samples were washed thrice with washing buffer for 8 h, and staining solution including 4′, 6-diamidino-2-phenylindole (DAPI, 1/1000 dilution, Sigma Aldrich), Alexa Fluor™ 568 Phalloidin (1/300 dilution, Life Technologies) and secondary e-cadherin antibody (1/200 dilution, Alexa Fluor™ 633 anti-rabbit) was prepared in washing buffer and added to hydrogels. Hydrogels were incubated in staining solution overnight at 4 °C on plate shaker. Once stained, samples were washed thrice with PBS for a total of 8 h and stored at 4 °C until imaging. ImageJ was used to quantify the number of nuclei per ROI per sample and determine the integrated density of nuclei, actin and e-cadherin channels per ROI.

### 4.20 Metabolic activity assay

The metabolic activity of cell-laden hydrogels was determined using a PrestoBlue® metabolic activity assay following the manufacturer's instructions. In brief, cell media was removed from the hydrogels and 400 μL of PrestoBlue® solution (1:10, PrestoBlue® reagent: cell media) was added to each hydrogel. Plates were then incubated in the PrestoBlue® solution in tissue culture incubator at 37 °C, 5 % CO2, 95 % air for 1 h. Following incubation, the solution from each well was removed and transferred in triplicate to a new 96-well plate in 100 μL volumes. Once PrestoBlue solution was removed, cell media was replenished in 24-well plates containing hydrogels and incubated in tissue culture incubators. Once transferred, the fluorescence of the 96-well plate was read at an excitation wavelength of 540 nm and emission wavelength of 590 nm using a CLARIOstar Plus spectrophotometer (BMG Labtech).

### 4.21 Drug treatment

A549s encapsulated in respective cell culture matrices were treated from day 3 to day 7 of culture with either dimethyl sulfoxide (DMSO, vehicle control) and 7 respective dose concentrations of doxorubicin hydrochloride solubilized in DMSO ranging from 1 × 10^−4^ μM–100 μM or ultra pure water (vehicle control) and 7 respective dose concentrations of cisplatin in ultra pure water ranging from 1 × 10^−3^ μM–1000 μM. Chemotherapeutic agents were administered on day 3 and day 5 of culture. Prior to addition of chemotherapeutic agents, media in wells was aspirated, and 200 μL of the appropriate concentration of drug was added per well. On day 7, the metabolic activity of cell-laden matrices was measured by PrestoBlue® assay and normalized to day 7 vehicle control. The IC_50_ each group was generated via [inhibitor] vs. response variable slope (four parameters) non-linear regression. n = 6. All data is represented as mean ± SD.

### 4.22 Statistical analysis

All statistical analyses were performed using GraphPad Prism v10. Technical replicates refer to individual hydrogel constructs within a single experiment, and biological replicates refer to independent experiments performed on separate days. Unless otherwise specified, data are presented as mean ± standard deviation. Normality of each dataset was assessed using the Shapiro Wilk test. For comparisons between two groups, normally distributed data were analyzed using unpaired Student's t tests with Welch's correction, and non-normally distributed data were analyzed using the Mann Whitney test. For comparisons involving three or more groups, parametric data were analyzed using one way ANOVA with Tukey's post hoc test, and non-parametric data were analyzed using the Kruskal–Wallis test with Dunn's multiple comparisons. For experiments involving two independent variables, two-way ANOVA with Tukey's post hoc test was employed. Outliers were removed only when objectively identified using the built in robust outlier detection in GraphPad Prism. Statistical tests used for each experiment are specified in the corresponding figure legends. Significance thresholds were defined as *∗p < 0.1*, *∗∗p < 0.01*, *∗∗∗p < 0.001*, *∗∗∗∗p < 0.0001*.

## Declaration of generative AI and AI-assisted technologies in the writing process

During the preparation of this work the authors used ChatGPT 4o in order to improve readability and grammar. After using this tool/service, the authors reviewed and edited the content as needed and takes full responsibility for the content of the published article.

## CRediT authorship contribution statement

**Luke Hipwood:** Data curation, Formal analysis, Methodology, Writing – original draft, Writing – review & editing. **Minne Dekker:** Data curation, Methodology, Writing – review & editing. **Dietmar W. Hutmacher:** Resources, Supervision, Writing – review & editing. **Christoph Meinert:** Conceptualization, Formal analysis, Funding acquisition, Resources, Supervision, Writing – review & editing. **Jacqui A. McGovern:** Conceptualization, Formal analysis, Funding acquisition, Project administration, Resources, Supervision, Writing – review & editing.

## Declaration of competing interest

The authors declare the following financial interests/personal relationships which may be considered as potential competing interests: Jacqui McGovern reports financial support was provided by The Kids Cancer Project. Christoph Meinert reports a relationship with Gelomics Pty Ltd that includes: employment and equity or stocks. Dietmar W Hutmacher reports a relationship with Gelomics Pty Ltd that includes: board membership, consulting or advisory, and equity or stocks. Luke Hipwood reports a relationship with Gelomics Pty Ltd that includes: employment. J.A.M.’s spouse is a cofounder, shareholder and employed by Gelomics Pty Ltd. C.M. is the Chief Executive Officer, a Co-Founder, Shareholder, and Executive Director of Gelomics Pty Ltd. D.W.H. is a Co-Founder, Shareholder, and member of the Scientific Advisory Board of Gelomics Pty Ltd. L.H. is employed by Gelomics Pty Ltd. Both L.H. and M.D. are PhD candidates co-supervised by Gelomics Pty Ltd. If there are other authors, they declare that they have no known competing financial interests or personal relationships that could have appeared to influence the work reported in this paper.

## Data Availability

Data are available from the corresponding authors upon reasonable request.
