# Supplementary material for: Photocrosslinkable lung dECM hydrogels promote stiffness-dependent lung cancer growth and chemoresistance
Source: Mater Today Bio. 2026 Jan 24;37:102838. doi: 10.1016/j.mtbio.2026.102838 (PMC12877845; doi:10.1016/j.mtbio.2026.102838)
Supplement: Multimedia component 1 [file mmc1.docx]

**Supplementary Material**

**Photocrosslinkable lung dECM hydrogels promote stiffness-dependent lung cancer growth and chemoresistance**

Luke Hipwood, Minne Dekker, Dietmar W. Hutmacher, Christoph Meinert* and Jacqui A. McGovern^*^

* Denotes shared corresponding authorship

Corresponding authors:

Christoph Meinert ([christoph.meinert@gelomics.com](mailto:christoph.meinert@gelomics.com)) and Jacqui A. McGovern ([jacqui.mcgovern@qut.edu.au](mailto:jacqui.mcgovern@qut.edu.au))


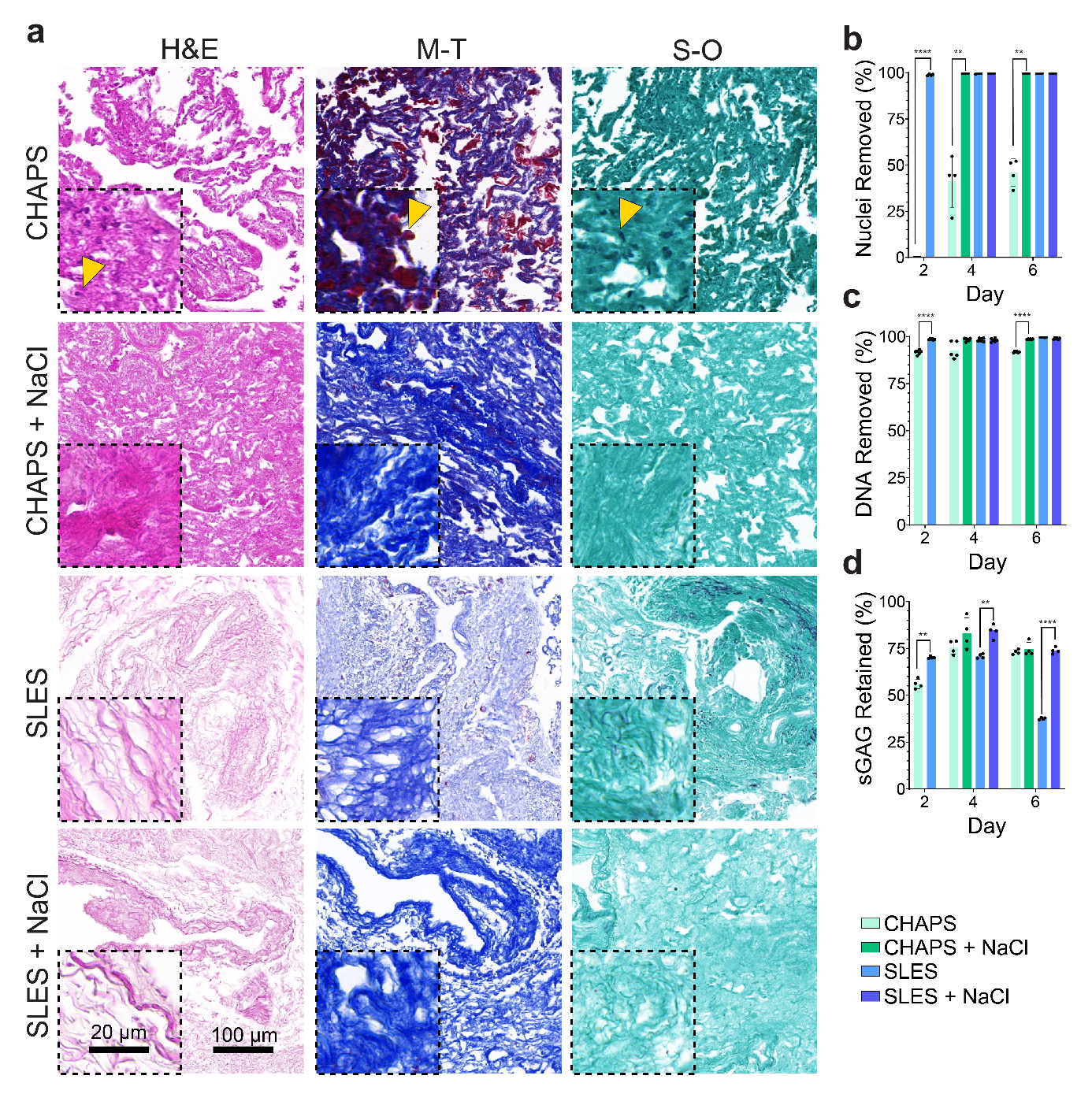


**Figure S1**. **Alternative detergent and sequential treatment methods for generation of lung dECMs.** **(A)** Histological staining of lung tissues on day 6 of decellularization revealed a lack of nuclei removal in CHAPS but retention of ECM signal in CHAPS conditions, and loss of both nuclei and ECM signal in SLES conditions. Yellow arrows = nuclei. Scale = 100 µm. Inset scale = 20 µm. **(B)** Nuclei removed over time, determined by quantification of tissue nuclei normalized to tissue area in ROI using ImageJ software. N =1. n = 4. **(C)** Lung dECM DNA content over time was quantified Quant-iT™ PicoGreen™ assay and normalized to sample weight. By day 6, dECMs generated through treatment via CHAPS + NaCl, SLES, or SLES + NaCl possessed a >99% removal of DNA. N = 1. n = 6. **(D)** The concentration of sGAGs in lung dECMs over time quantified by DMMB assay and normalized to sample weight. By day 6, dECMs prepared through treatment with SLES exhibited the lowest sGAG content from the groups. N = 1. n = 4. Mann Whitney tests were used to analyze day 2 samples, while non-parametric ANOVA tests were used to analyze day 4 and day 6 samples. All data is represented as mean ± SD. **p* < 0.1, ***p* < 0.01, ****p* < 0.001, *****p* < 0.0001.


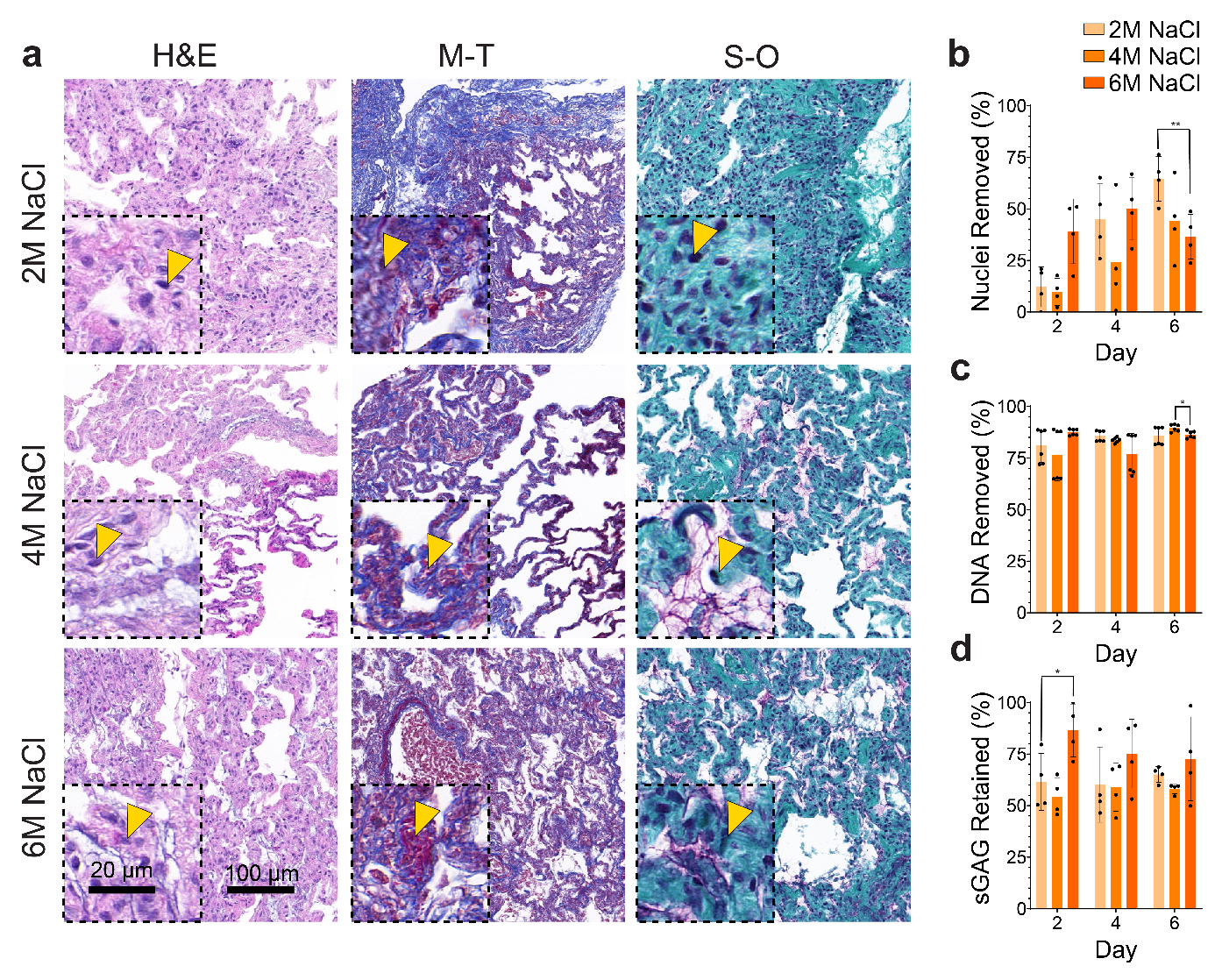


**Figure S2. Alternative hypertonic decellularization methods are ineffective for complete decellularization of porcine lung tissue.** **(A)** Histological staining of lung tissues on day 6 of decellularization revealed a lack of nuclei removal in all conditions tested, and potential retention of salt crystals despite extensive washing. Yellow arrows = nuclei. Scale = 100 µm. Inset scale = 20 µm. **(B)** Nuclei removed over time, determined by quantification of tissue nuclei normalized to tissue area in ROI using ImageJ software. N = 1. n = 4. **(C)** Lung dECM DNA content over time was quantified Quant-iT™ PicoGreen™ assay and normalized to sample weight. By day 6, all dECMs presented with ~80% DNA removal compared to native tissue control. N = 1. n = 6. **(D)** The concentration sGAGs in lung dECMs over time quantified by DMMB assay and normalized to sample weight. By day 6, all dECMs presented with 55-70% retention of sGAGs compared to native tissue control. N = 1. n = 4. Mann Whitney tests were used to analyze day 2 samples, while non-parametric ANOVA tests were used to analyze day 4 and day 6 samples. n = 4-6. All data is represented as mean ± SD. **p* < 0.1, ***p* < 0.01.

**Table S1.** DNA content of lung dECMs treated with detergents over time*

|  | Triton X-100 | STDEV (± ng/mg) | SLES | STDEV (± ng/mg) | CHAPS | STDEV (± ng/mg) |
| --- | --- | --- | --- | --- | --- | --- |
| Day 2 | 213.12 | 35.97 | 22.33 | 5.06 | 130.44 | 14.7 |
| Day 4 | 219.33 | 36.33 | 26.23 | 13.3 | 157.9 | 18.77 |
| Day 6 | 111.28 | 33.72 | 5.4 | 1.26 | 124.67 | 5.19 |

*Note: The DNA content of native lung ECM (day 0) was measured as 4,819.33 ± 619.53 ng/mg.

**Table S2.** DNA content of lung dECMs treated with hypertonic solutions over time

|  | 1M NaCl | STDEV (± ng/mg) | 2M NaCl | STDEV (± ng/mg) | 4M NaCl | STDEV (± ng/mg) | 6M NaCl | STDEV (± ng/mg) |
| --- | --- | --- | --- | --- | --- | --- | --- | --- |
| Day 2 | 327.25 | 83.47 | 286.71 | 123.78 | 356.3 | 190.77 | 189.25 | 23.48 |
| Day 4 | 299.13 | 91.72 | 216.61 | 39.94 | 250.07 | 18.87 | 212.64 | 3.16 |
| Day 6 | 93.8 | 11.64 | 215.7 | 66.44 | 154.8 | 26.02 | 206.02 | 26.16 |

**Table S3.** DNA content of lung dECMs treated with detergents and hypertonic solutions over time

|  | Triton X-100 + NaCl | STDEV (± ng/mg) | SLES + NaCl | STDEV (± ng/mg) | CHAPS + NaCl | STDEV (± ng/mg) |
| --- | --- | --- | --- | --- | --- | --- |
| Day 2 | 213.12 | 35.97 | 22.33 | 5.06 | 125.95 | 17.13 |
| Day 4 | 36.46 | 10.41 | 29.34 | 13.96 | 25.88 | 12.56 |
| Day 6 | 15.29 | 2.39 | 16.25 | 5.69 | 20.88 | 1.38 |


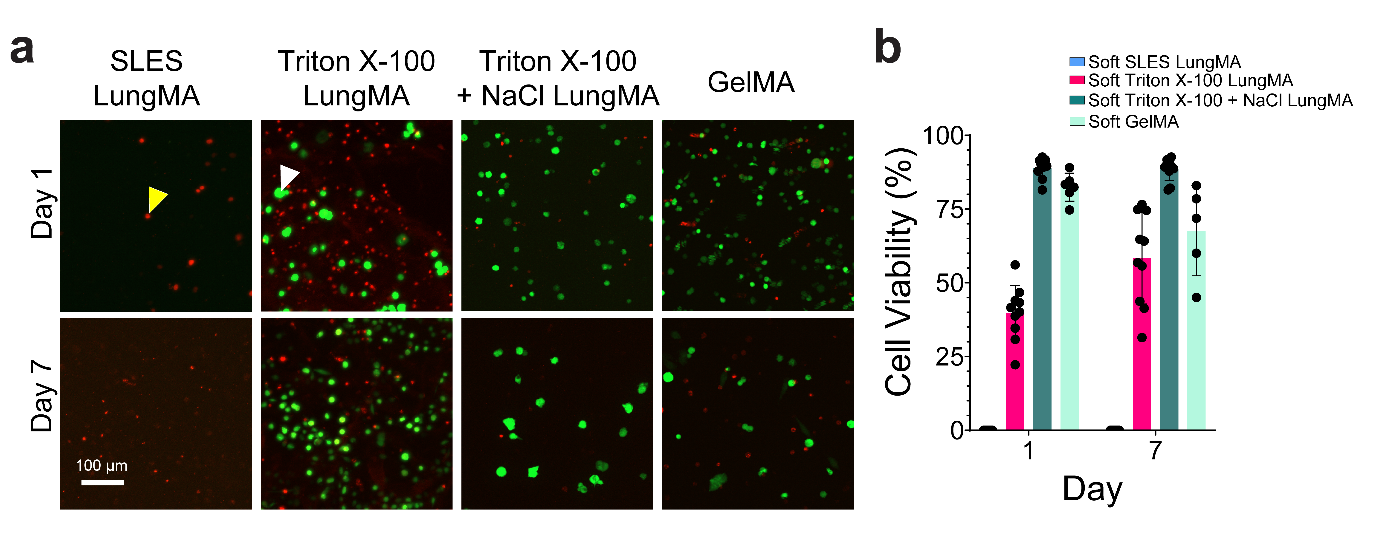


**Figure S3. Viability of SAOS-2 cells cultured in various LungMA matrices and GelMA at day 1 and day 7 of culture.** SAOS-2 cells (P20, ATCC) were encapsulated in soft LungMA and GelMA hydrogels (30 µL volume) at a seeding density of 1 × 10^6^ cells/mL and cultured in McCoy’s media (Gibco™) + 10% FBS + 1% P/S, with complete media changes every 2 days post-encapsulation. **(A)** Viability staining of encapsulated A549 cells using FDA (green, live) and PI (red, dead) revealed low cell viability in SLES and Triton X-100 hydrogels over time. Images captured using Zeiss Axio Observer at 10 × magnification. Yellow arrow = dead cell. White arrow = live cell. Scale = 500 µm. Inset scale = 200 µm. **(B)** Viability quantification. The viability of encapsulated SAOS-2 cells was determined by ImageJ quantification of live (green) and dead (red) channels of maximum projection Z-stacks. N = 1. n = 5-10. All data is represented as mean ± SD.


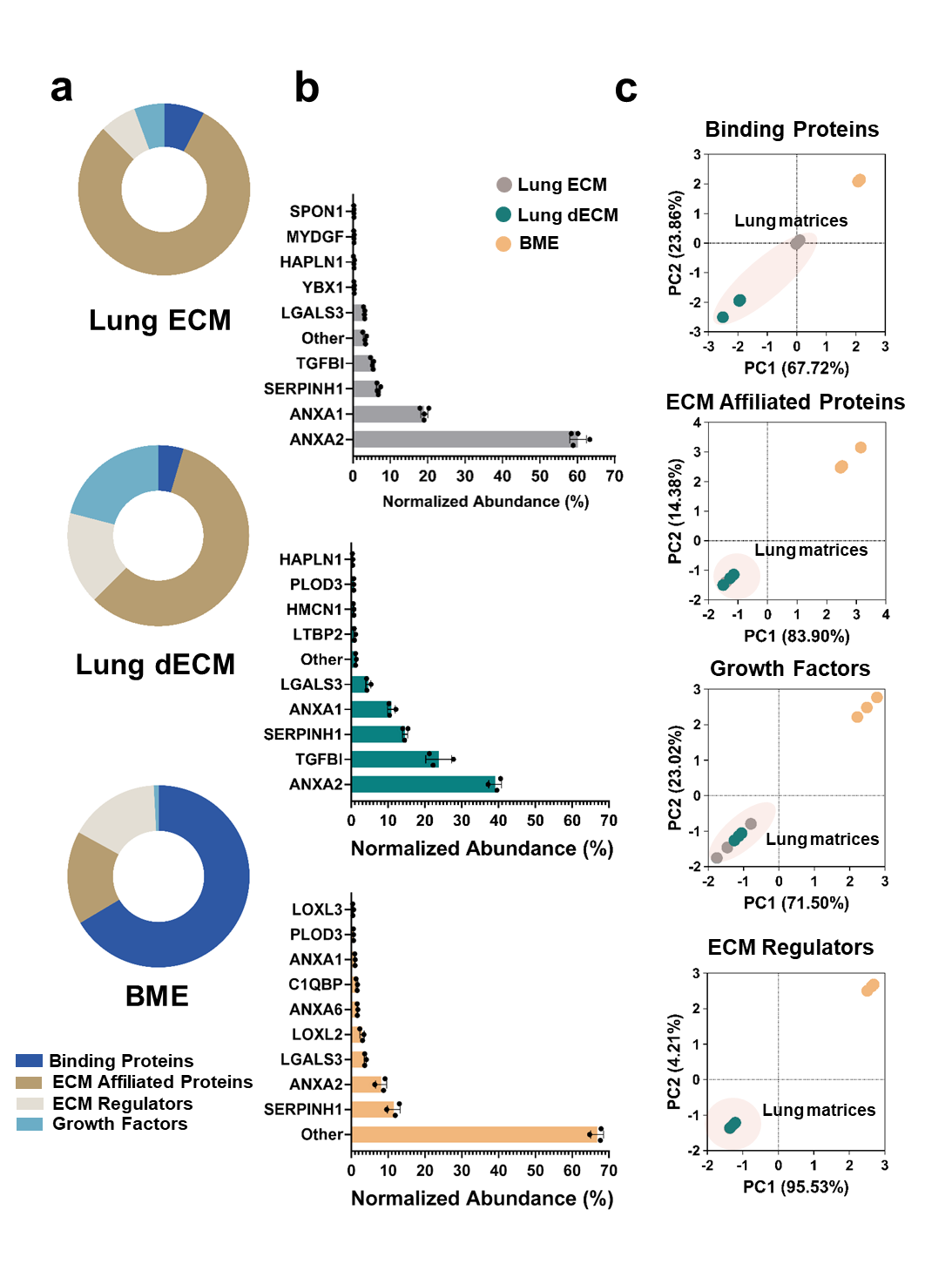


**Figure S4. Proteomic analyzes of porcine lung ECM, dECM, and BME reveals conservation of lung ECM binding proteins, affiliated, regulators and growth factors post-decellularization.** The abundance of proteins was normalized against total protein abundance within each group to determine protein distribution and rank. Principal core analyzes (PCA) of protein profiles of lung ECM, dECM, and BME samples displayed a conservation of proteins between the lung ECM and dECM groups. **(A)** Distribution of matrisome affiliated proteins in lung ECM, lung dECM and BME. **(B)** Normalized abundance of the 10 most abundant matrisome affiliated proteins within lung ECM, lung dECM, and BME. n = 3-4. All data is represented as mean ± SD. **(C)** PCA of matrisome-affiliated proteins, binding proteins, ECM regulators, and growth factors. N = 1. n = 3-4.

**Table S4.** Percentage of matrisome proteins present in lung ECM, dECM and BME

| Protein(s) | Average Lung ECM (%) | STDEV Lung ECM | Average Lung dECM (%) | STDEV Lung dECM | Average BME (%) | STDEV BME |
| --- | --- | --- | --- | --- | --- | --- |
| Collagens | 20.33 | 5.17 | 48.50 | 7.42 | 0.66 | 0.06 |
| Fibronectin | 2.88 | 0.30 | 3.83 | 0.67 | 0.41 | 0.06 |
| Glycoproteins | 3.98 | 1.04 | 4.90 | 0.63 | 0.40 | 0.06 |
| Laminins | 7.76 | 0.63 | 7.18 | 1.39 | 92.25 | 4.23 |
| Proteoglycans | 13.25 | 4.72 | 14.89 | 4.29 | 3.50 | 0.57 |
| Vimentin | 18.60 | 2.80 | 13.89 | 3.31 | 1.83 | 0.133 |
| Binding Proteins | 1.32 | 0.22 | 0.19 | 0.03 | 0.66 | 0.03 |
| ECM Affiliated Proteins | 27.60 | 2.912 | 3.92 | 0.68 | 0.15 | 0.02 |
| ECM Regulators | 2.46 | 0.07 | 1.10 | 0.16 | 0.14 | 0.03 |
| Growth Factors | 1.84 | 0.14 | 1.62 | 0.09 | 0.01 | 0.01 |

**Table S5.1** Relative abundance of collagen subtypes expressed as % of total collagen present in lung ECM, dECM and BME

| Collagen | Mean relative abundance Lung ECM (% of total collagen) | STDEV Lung ECM | Mean relative abundance Lung dECM (% of total collagen) | STDEV Lung dECM | Mean relative abundance BME (% of total collagen) | STDEV BME |
| --- | --- | --- | --- | --- | --- | --- |
| COL1A1 | 4.47 | 3.00 | 0.23 | 0.05 | 0.73 | 0.09 |
| COL1A2 | 1.48 | 0.22 | 0.36 | 0.07 | 0.07 | 0.01 |
| COL2A1 | 0.28 | 0.09 | 0.02 | - | - | - |
| COL3A1 | - | - | - | - | 0.05 | 0.00 |
| COL4A1 | 0.43 | 0.15 | 0.32 | 0.26 | 15.58 | 1.48 |
| COL4A2 | 0.02 | 0.00 | 0.02 | 0.05 | 50.77 | 2.14 |
| COL4A3 | 0.05 | 0.01 | 0.04 | 0.01 | - | - |
| COL4A4 | 0.03 | 0.01 | 0.04 | 0.01 | - | - |
| COL4A6 | 0.05 | - | 0.07 | 0.01 | - | - |
| COL5A1 | 0.23 | 0.06 | 0.07 | 0.02 | - | - |
| COL5A2 | 0.05 | 0.01 | 0.02 | - | - | - |
| COL6A1 | 26.34 | 3.37 | 35.37 | 1.90 | 0.91 | 0.12 |
| COL6A2 | 19.06 | 3.26 | 27.63 | 0.55 | 0.44 | 0.03 |
| COL6A3 | 22.51 | 2.75 | 29.99 | 1.87 | 4.19 | 0.49 |
| COL6A5 | 0.97 | 0.18 | 1.18 | 0.23 | - | - |
| COL6A6 | 1.11 | 0.14 | 1.75 | 0.22 | - | - |
| COL10A1 | 0.01 | - | - | - | - | - |
| COL11A1 | 0.38 | 0.09 | 0.07 | 0.05 | - | - |
| COL11A2 | 0.14 | 0.02 | - | - | - | - |
| COL12A1 | 6.51 | 0.34 | 0.91 | 0.22 | 1.59 | 0.15 |
| COL14A1 | 12.66 | 7.88 | 0.93 | 0.24 | 12.17 | 0.81 |
| COL15A1 | 2.34 | 0.45 | 0.74 | 0.22 | 6.69 | 1.62 |
| COL18A1 | 0.88 | 0.30 | 0.25 | 0.07 | 6.71 | 0.41 |

**Table S5.2** Percentage of collagens present in lung ECM, dECM and BME relative to total ECM

| Collagen | Average Lung ECM (%) | STDEV Lung ECM | Average Lung dECM (%) | STDEV Lung dECM | Average BME (%) | STDEV BME |
| --- | --- | --- | --- | --- | --- | --- |
| COL1A1 | 0.945230769 | 0.657068417 | 0.109766514 | 0.017676514 | 0.004856061 | 0.00079638 |
| COL1A2 | 0.297080155 | 0.017986252 | 0.171542942 | 0.014604602 | 0.000493013 | 0.000104191 |
| COL2A1 | 0.05608141 | 0.017611527 | 0.009048613 | 0.00124539 | - | - |
| COL3A1 | - | - | - | - | 0.000338329 | 4.58887E-05 |
| COL4A1 | 0.085063325 | 0.025450368 | 0.157236074 | 0.118356184 | 0.103301268 | 0.014807193 |
| COL4A2 | 0.003729814 | 0.000594065 | 0.009549648 | 0.001665858 | 0.335617215 | 0.022834004 |
| COL4A3 | 0.010084133 | 0.001465302 | 0.016638318 | 0.004862339 | - | - |
| COL4A4 | 0.006585964 | 0.001002162 | 0.019686393 | 0.005096596 | - | - |
| COL4A6 | 0.010825937 | 0.001888325 | 0.036630171 | 0.010019579 | - | - |
| COL5A1 | 0.045753622 | 0.007739988 | 0.032229555 | 0.006766401 | - | - |
| COL5A2 | 0.01079046 | 0.002171308 | 0.007839598 | 0.003179253 | - | - |
| COL6A1 | 5.34609419 | 0.878698002 | 17.11450367 | 2.006159766 | 0.0060014 | 0.0010512 |
| COL6A2 | 3.871879488 | 0.805634184 | 13.38144956 | 1.684316614 | 0.002899321 | 8.51005E-05 |
| COL6A3 | 4.569717394 | 0.723509738 | 14.61248887 | 2.88310776 | 0.027628221 | 0.002728242 |
| COL6A5 | 0.19610258 | 0.039874165 | 0.573950658 | 0.146285209 | - | - |
| COL6A6 | 0.224553213 | 0.02831639 | 0.857833572 | 0.238261695 | - | - |
| COL10A1 | 0.001865663 | 0.000163005 | - | - | - | - |
| COL11A1 | 0.076805791 | 0.010315744 | 0.034143867 | 0.021925726 | - | - |
| COL11A2 | 0.027970367 | 0.001601838 | - | - | - | - |
| COL12A1 | 1.320363914 | 0.128703407 | 0.437225793 | 0.075747799 | 0.011116945 | 0.000551443 |
| COL14A1 | 2.576485663 | 1.689367076 | 0.445174585 | 0.079646282 | 0.080473662 | 0.00759595 |
| COL15A1 | 0.473640685 | 0.094097162 | 0.353033536 | 0.077866565 | 0.043874714 | 0.008847412 |
| COL18A1 | 0.174432737 | 0.038634546 | 0.117684862 | 0.026256096 | 0.044344463 | 0.002639149 |

**Table S6.1** Relative abundance of proteoglycan subtypes expressed as % of total proteoglycan present in lung ECM, dECM and BME

| Proteoglycan | Mean relative abundance Lung ECM (% of total proteoglycan) | STDEV Lung ECM | Mean relative abundance Lung dECM (% of total proteoglycan) | STDEV Lung dECM | Mean relative abundance BME (% of total proteoglycan) | STDEV BME |
| --- | --- | --- | --- | --- | --- | --- |
| AGRN | 4.38 | 1.46 | 8.57 | 3.07 | 2.10 | 0.38 |
| BGN | 15.36 | 6.18 | 26.41 | 4.09 | 0.83 | 0.21 |
| DCN | 15.57 | 3.78 | 17.36 | 3.42 | - | - |
| FMOD | 2.73 | 0.20 | 1.01 | 0.02 | - | - |
| GPC6 | 0.10 | 0.02 | - | - | - | - |
| HSPG2 | 21.45 | 6.36 | 24.01 | 7.50 | 96.52 | 0.66 |
| KERA | - | - | 0.05 | 0.01 | - | - |
| LUM | 21.2 | 4.04 | 5.98 | 0.92 | 0.55 | 0.08 |
| VCAN | 3.02 | 2.10 | 0.72 | 0.24 | - | - |
| OGN | 8.51 | 2.42 | 14.01 | 3.23 | - | - |
| ASPN | 2.62 | 0.54 | 1.48 | 0.48 | - | - |
| ACAN | 5.96 | 1.70 | 0.39 | 0.02 | - | - |
|  |  |  |  |  |  |  |

**Table S6.2** Percentage of proteoglycans present in lung ECM, dECM and BME relative total ECM

| Proteoglycan | Average Lung ECM (%) | STDEV Lung ECM | Average Lung dECM (%) | STDEV Lung dECM | Average BME (%) | STDEV BME |
| --- | --- | --- | --- | --- | --- | --- |
| AGRN | 0.55653611 | 0.101152772 | 1.238809745 | 0.309553366 | 0.0720681 | 0.002606134 |
| BGN | 2.145478827 | 1.329496031 | 3.965019496 | 1.083035725 | 0.028456895 | 0.00280474 |
| DCN | 2.127374041 | 0.94454186 | 2.641345265 | 0.993217439 | - | - |
| FMOD | 0.212641502 | 0.184349612 | 0.150903693 | 0.027111507 | - | - |
| GPC6 | 0.013015859 | 0.000506126 | - | - | - | - |
| HSPG2 | 2.726525453 | 0.348959369 | 3.46014663 | 0.629458878 | 3.385457461 | 0.563678999 |
| KERA | - | - | 0.007761789 | 0.000553808 | - | - |
| LUM | 2.87751201 | 1.128674072 | 0.905916983 | 0.298877047 | 0.018999528 | 0.002719703 |
| VCAN | 0.37350697 | 0.210834235 | 0.103125619 | 0.021017917 | - | - |
| OGN | 1.109252398 | 0.272936534 | 2.13315676 | 0.826454953 | - | - |
| ASPN | 0.346217273 | 0.091017465 | 0.22380639 | 0.087682934 | - | - |
| ACAN | 0.7638833 | 0.109045391 | 0.056918015 | 0.008445233 | - | - |

**Table S7.** Relative abundance of laminin subtypes expressed as % of total laminin present in lung ECM, dECM and BME

| Laminin | Mean relative abundance Lung ECM (% of total laminin) | STDEV Lung ECM | Mean relative abundance Lung dECM (% of total laminin) | STDEV Lung dECM | Mean relative abundance BME (% of total laminin) | STDEV BME |
| --- | --- | --- | --- | --- | --- | --- |
| LAMA1 | - | - | - | - | 44.38 | 2.23 |
| LAMA2 | 1.14 | 0.06 | 2.56 | 0.04 | 0.01 | 0.00 |
| LAMA3 | 11.34 | 1.79 | 13.19 | 0.55 | - | - |
| LAMA4 | 0.35 | 0.06 | 0.93 | 0.14 | 0.01 | 0.00 |
| LAMA5 | 1.32 | 0.38 | 4.16 | 0.02 | 0.13 | 0.01 |
| LAMB1 | 6.56 | 0.30 | 5.53 | 0.47 | 29.37 | 0.27 |
| LAMB2 | 1.553 | 0.13 | 5.46 | 0.47 | 0.10 | 0.00 |
| LAMB3 | 64.46 | 2.24 | 39.55 | 0.56 | - | - |
| LAMC1 | 0.06 | 0.02 | 0.66 | 0.11 | 26.00 | 2.01 |
| LAMC2 | 13.21 | 1.58 | 27.95 | 0.95 | - | - |

**Table S7.2** Percentage of laminins present in lung ECM, dECM and BME relative to total ECM

| Laminin | Average Lung ECM (%) | STDEV Lung ECM | Average Lung dECM (%) | STDEV Lung dECM | Average BME (%) | STDEV BME |
| --- | --- | --- | --- | --- | --- | --- |
| LAMA1 | - | - | - | - | 40.93432726 | 1.859827139 |
| LAMA2 | 0.088193221 | 0.007993034 | 0.184100514 | 0.037823988 | 0.005142396 | 0.000349364 |
| LAMA3 | 0.881976274 | 0.157349054 | 0.943851383 | 0.169745134 | - | - |
| LAMA4 | 0.027229181 | 0.003594713 | 0.067194682 | 0.018006526 | 0.013100389 | 0.000887137 |
| LAMA5 | 0.102463343 | 0.030255897 | 0.298343339 | 0.05648704 | 0.118699095 | 0.006952986 |
| LAMB1 | 0.509574314 | 0.032750778 | 0.399780585 | 0.105269972 | 27.09316232 | 0.3618087 |
| LAMB2 | 0.12081919 | 0.015039404 | 0.392248034 | 0.078396945 | 0.092578091 | 0.001986896 |
| LAMB3 | 5.003501819 | 0.299302384 | 2.842526977 | 0.578051543 | - | - |
| LAMC1 | 0.004793234 | 0.001988776 | 0.047418395 | 0.010445073 | 23.98865734 | 1.984609472 |
| LAMC2 | 1.022197814 | 0.084909205 | 1.999629562 | 0.337629088 | - | - |

**Table S8.1** Relative abundance of ECM binding proteins expressed as % of total ECM binding proteins present in lung ECM, dECM and BME

| ECM binding protein | Mean relative abundance Lung ECM (% of total ECM binding proteins) | STDEV Lung ECM | Mean relative abundance Lung dECM (% of total ECM binding proteins) | STDEV Lung dECM | Mean relative abundance BME (% of total ECM binding proteins) | STDEV BME |
| --- | --- | --- | --- | --- | --- | --- |
| C1QBP | 2.43 | 0.32 | 2.27 | 0.60 | 2.21 | 0.27 |
| HSBP1 | 4.56 | 0.61 | 6.94 | 1.16 | 0.14 | 0.03 |
| LTBP2 | 3.09 | 0.28 | 35.32 | 6.91 | - | - |
| YBX1 | 8.63 | 0.22 | 9.21 | 3.24 | 0.55 | 0.08 |
| Other | 81.28 | 0.48 | 46.25 | 3.51 | 97.11 | 0.27 |

**Table S8.2** Percentage of ECM binding proteins present in lung ECM, dECM and BME relative to total ECM

| ECM binding protein | Average Lung ECM (%) | STDEV Lung ECM | Average Lung dECM (%) | STDEV Lung dECM | Average BME (%) | STDEV BME |
| --- | --- | --- | --- | --- | --- | --- |
| C1QBP | 0.032313557 | 0.008342457 | 0.004253083 | 0.001402106 | 0.01459596 | 0.002234075 |
| HSBP1 | 0.059774738 | 0.010783898 | 0.013018463 | 0.003604971 | 0.000890083 | 0.000159912 |
| LTBP2 | 0.041111051 | 0.010594915 | 0.064420878 | 0.005244281 | - | - |
| YBX1 | 0.11381373 | 0.020675291 | 0.0175231 | 0.008398696 | 0.003621105 | 0.000616119 |
| Other | 1.069274715 | 0.170261314 | 0.085960221 | 0.015134656 | 0.639924213 | 0.023696163 |

**Table S9.1** Relative abundance of matrisome-affiliated proteins expressed as % of total matrisome proteins present in lung ECM, dECM and BME

| ECM-affiliated protein | Mean relative abundance Lung ECM (% of total matrisome-affiliated proteins) | STDEV Lung ECM | Mean relative abundance Lung dECM (% of total matrisome-affiliated proteins) | STDEV Lung dECM | Mean relative abundance BME (%of total matrisome-affiliated proteins) | STDEV BME |
| --- | --- | --- | --- | --- | --- | --- |
| ANXA1 | 23.01 | 1.71 | 19.33 | 1.13 | 6.64 | 0.49 |
| ANXA2 | 72.48 | 2.06 | 69.22 | 2.15 | 53.04 | 4.07 |
| ANXA6 | - | - | - | - | 11.21 | 1.39 |
| HMCN1 | 0.01 | - | 1.22 | 0.23 | - | - |
| LGALS3 | 3.72 | 0.28 | 8.14 | 0.90 | 25.19 | 2.31 |
| PLOD3 | 0.32 | 0.05 | 1.21 | 0.04 | 3.92 | 0.31 |
| SPON1 | 0.34 | 0.04 | 0.19 | 0.03 | - | - |
| VIT | 0.13 | 0.01 | 0.69 | 0.19 | - | - |

**Table S9.2** Percentage of matrisome-affiliated proteins present in lung ECM, dECM and BME relative to total ECM

| ECM-affiliated protein | Average Lung ECM (%) | STDEV Lung ECM | Average Lung dECM (%) | STDEV Lung dECM | Average BME (%) | STDEV BME |
| --- | --- | --- | --- | --- | --- | --- |
| ANXA2 | 20.0110254 | 2.164369442 | 2.71118917 | 0.464699284 | 0.077667614 | 0.013470785 |
| ANXA1 | 6.337933443 | 0.638727253 | 0.757176177 | 0.136761155 | 0.009623226 | 0.000350676 |
| ANXA6 | - | - | - | - | 0.016207513 | 0.000942286 |
| LGALS3 | 1.022572939 | 0.078716276 | 0.319241898 | 0.067916256 | 0.036504392 | 0.002110048 |
| VIT | 0.034884994 | 0.001174052 | 0.026040736 | 0.002544205 | - | - |
| HMCN1 | 0.00356427 | 0.000122299 | 0.04697659 | 0.004564316 | - | - |
| SPON1 | 0.094454042 | 0.016012189 | 0.007665173 | 0.002184564 | - | - |
| PLOD3 | 0.087386103 | 0.012760035 | 0.047213277 | 0.006327202 | 0.005681437 | 0.000204037 |

**Table S10.1** Relative abundance of ECM regulators expressed as % of total ECM regulators present in lung ECM, dECM and BME

| ECM regulator | Mean relative abundance Lung ECM (% of total ECM regulators) | STDEV Lung ECM | Mean relative abundance Lung dECM (%of total ECM regulators) | STDEV Lung dECM | Mean relative abundance BME (%of total ECM regulators) | STDEV BME |
| --- | --- | --- | --- | --- | --- | --- |
| C1QTNF5 | 0.22 | 0.05 | 0.31 | 0.04 | - | - |
| HAPLN1 | 3.90 | 1.33 | 2.52 | 0.42 | - | - |
| HAPLN3 | 0.68 | 0.08 | 0.34 | 0.07 | - | - |
| LOX | - | - | 1.92 | 0.88 | - | - |
| LOXL1 | - | - | 1.06 | 0.15 | 0.78 | 0.03 |
| LOXL2 | 0.24 | 0.03 | 0.30 | 0.05 | 19.74 | 5.46 |
| LOXL3 | - | - | 0.08 | 0.01 | 3.28 | 0.64 |
| SERPINB1 | 0.72 | 0.07 | 0.30 | 0.03 | - | - |
| SERPINB10 | 0.81 | 0.08 | - | - | - | - |
| SERPINB8 | 1.93 | 0.43 | 1.20 | 0.38 | - | - |
| SERPINH1 | 91.49 | 1.10 | 91.97 | 0.91 | 76.21 | 4.96 |
|  |  |  |  |  |  |  |

**Table S10.2** Percentage of ECM regulators present in lung ECM, dECM and BME relative to total ECM

| ECM regulator | Average Lung ECM (%) | STDEV Lung ECM | Average Lung dECM (%) | STDEV Lung dECM | Average BME (%) | STDEV BME |
| --- | --- | --- | --- | --- | --- | --- |
| C1QTNF5 | 0.00545068 | 0.001237704 | 0.00337904 | 0.00052812 | - | - |
| HAPLN1 | 0.096026244 | 0.033303446 | 0.027498054 | 0.004807651 | - | - |
| HAPLN3 | 0.01678786 | 0.001920618 | 0.003710324 | 0.000476226 | - | - |
| LOX | - | - | 0.021580631 | 0.01196217 | - | - |
| LOXL2 | 0.005903015 | 0.00068356 | 0.003244685 | 0.000478673 | 0.028107451 | 0.005558114 |
| LOXL3 | - | - | 0.000869025 | 9.61148E-05 | 0.004761367 | 0.00120861 |
| LOXL1 | - | - | 0.011516011 | 0.001432977 | 0.00112376 | 9.1457E-05 |
| SERPINH1 | 2.247492211 | 0.016133237 | 1.010653276 | 0.135515887 | 0.11061103 | 0.016653021 |
| SERPINB8 | 0.047246557 | 0.010287808 | 0.012866861 | 0.002243344 | - | - |
| SERPINB1 | 0.01781095 | 0.001848385 | 0.003261347 | 0.00023454 | - | - |
| SERPINB10 | 0.019920542 | 0.002189253 | - | - | - | - |

**Table S11.1** Relative fraction of growth factors within growth factor group present in lung ECM, dECM and BME

| Growth Factor | Average Lung ECM (%) | STDEV Lung ECM | Average Lung dECM (%) | STDEV Lung dECM | Average BME (%) | STDEV BME |
| --- | --- | --- | --- | --- | --- | --- |
| EFEMP2 | - | - | - | - | 77.79 | 2.57 |
| MYDGF | 5.16 | 0.56 | 0.07 | - | 22.21 | 2.57 |
| TGFB1 | 94.68 | 0.56 | 99.83 | 0.01 | - | - |
| TGFB2 | 0.15 | - | 0.1 | 0.01 | - | - |

**Table S11.2** Percentage of growth factors present in lung ECM, dECM and BME relative to total ECM

| Growth Factor | Average Lung ECM (%) | STDEV Lung ECM | Average Lung dECM (%) | STDEV Lung dECM | Average BME (%) | STDEV BME |
| --- | --- | --- | --- | --- | --- | --- |
| EFEMP2 | - | - | - | - | 0.006837767 | 0.001372417 |
| MYDGF | 0.095349813 | 0.018007416 | 0.001161881 | 0.000196464 | 0.001981296 | 0.000634082 |
| TGFBI | 1.737702608 | 0.126224777 | 1.621304607 | 0.08949061 | - | - |
| TGFB2 | 0.00275713 | 0.000207384 | 0.001608863 | 9.73531E-05 | - | - |

**Table S12.1.** Previous reports of the stiffness of lung tissues^*^

| Animal of Origin | Sample Type | Matrix Type | Testing Method(s) | Stiffness Measurement | Stiffness | Reference |
| --- | --- | --- | --- | --- | --- | --- |
| Human | Tissue | Healthy lung tissue | Sequential stress relaxation (SSR) | Stiffness | ~7.5 N/m | [22] |
| Human | Tissue | Idiopathic pulmonary fibrosis (IPF) lung tissue | SSR | Stiffness | ~40 N/m | [22] |
| Human | Tissue | Decellularized healthy lung tissue | SSR | Stiffness | ~ 20 N/m | [22] |
| Human | Tissue | Decellularized IPF lung tissue | SSR | Stiffness | ~37.5 N/m | [22] |
| Human | Tissue | Healthy lung tissue | Low load compression testing (LLCT) | Young's modulus | ~ 3 kPa | [26] |
| Human | Tissue | Chronic obstructive pulmonary disorder (COPD) lung tissue | LLCT | Young's modulus | 3 kPa | [26] |
| Human | Tissue | IPF lung tissue | LLCT | Young's modulus | ~18 kPa | [26] |
| Human | Tissue | Healthy lung tissue | Atomic force microscopy (AFM) | Young's modulus | 2 kPa | [112] |
| Human | Tissue | IPF lung tissue | AFM | Young's modulus | 10 kPa | [112] |
| Human | Tissue | Healthy lung decellularized extracellular matrix (dECM) | AFM | Young's modulus | 1.6 kPa | [112] |
| Human | Tissue | IPF lung dECM | AFM | Young's modulus | 8 kPa | [112] |
| Human | Tissue | 11-30 yrs human lung airway tissue | AFM | Young's modulus | ~10 kPa | [203] |
| Human | Tissue | 41-60 yrs human lung airway tissue | AFM | Young's modulus | ~ 10 kPa | [203] |
| Human | Tissue | 11-30 yrs human lung vessel tissue | AFM | Young's modulus | ~ 5 kPa | [203] |
| Human | Tissue | 41-60 yrs human lung vessel tissue | AFM | Young's modulus | ~9 kPa | [203] |
| Human | Tissue | 11-30 yrs human lung parenchyma tissue | AFM | Young's modulus | ~1.5 kPa | [203] |
| Human | Tissue | 41-60 yrs human lung parenchyma vessel tissue | AFM | Young's modulus | ~2.19 kPa | [203] |
| Porcine | Tissue | Lung tissue | Compression testing, 5% strain | Young's modulus | ~8.52 kPa | [40] |
| Porcine | Tissue | Lung tissue | Compression testing, 30% strain | Young's modulus | ~77 kPa | [40] |
| Porcine | Tissue | Lung dECM | Compression testing, 5% strain | Young's modulus | ~ 7 kPa | [40] |
| Porcine | Tissue | Lung dECM | Compression testing, 30% strain | Young's modulus | ~ 67 kPa | [40] |
| Porcine | Tissue | Triton X-100/ sodium deoxycholate (SDC)-treated lung dECM | Compression testing, 5% strain | Young's modulus | ~ 5.4 kPa | [40] |
| Porcine | Tissue | Triton X-100/SDC-treated lung dECM | Compression testing, 30% strain | Young's modulus | ~ 54 kPa | [40] |
| Porcine | Tissue | Sodium dodecyl sulfate (SDS)-treated lung dECM | Compression testing, 5% strain | Young's modulus | ~ 9.28 kPa | [40] |
| Porcine | Tissue | SDS lung dECM | Compression testing, 30% strain | Young's modulus | ~ 143 kPa | [40] |

^*^Stiffness values across studies should be interpreted with caution due to differences in testing protocols, including strain parameters.

**Table S12.2.** Previous reports of the stiffness of lung-derived hydrogels^*^

| Animal of Origin | Sample Type | Matrix Type | Concentrations | Testing Method(s) | Stiffness Measurement | Stiffness | Reference number |
| --- | --- | --- | --- | --- | --- | --- | --- |
| Bovine | Hydrogel | Iodine/freeze-thaw lung dECM | 15 mg/mL lung dECM | Rheology | Storage modulus | 200 Pa | [204] |
| Bovine | Hydrogel | Peracetic acid/Triton X-100/SDC lung dECM | 15 mg/mL lung dECM | Rheology | Storage modulus | 1 Pa | [204] |
| Bovine | Hydrogel | SDS lung dECM | 15 mg/mL lung dECM | Rheology | Storage modulus | 50 Pa | [204] |
| Bovine | Hydrogel | Triton X-100 lung dECM | 15 mg/mL lung dECM | Rheology | Storage modulus | 110 Pa | [204] |
| Bovine | Hydrogel | Lung dECM | 15 mg/L lung dECM | Rheology | Storage modulus | 200 Pa | [204] |
| Bovine | Hydrogel | Lung dECM | 15 mg/mL lung dECM | Rheology | Storage modulus | ~75 Pa | [205] |
| Human | Hydrogel | Alveolar dECM | 30 mg/mL alveolar dECM | AFM | Young's modulus | 52 Pa | [17] |
| Human | Hydrogel | Alveolar dECM | 12 mg/mL alveolar dECM | AFM | Young's modulus | ~25 Pa | [17] |
| Human | Hydrogel | Alveolar dECM | 8 mg/mL alveolar dECM | AFM | Young's modulus | 10.5 Pa | [17] |
| Human | Hydrogel | Alveolar dECM | 4 mg/mL alveolar dECM | AFM | Young's modulus | N/A | [17] |
| Human | Cell-laden hydrogel | Healthy lung dECM | Non-IPF fibroblast-laden 20 mg/mL healthy lung dECM hydrogels, day 7 of culture | LLCT | Young's modulus | ~3.5 kPa | [23] |
| Human | Cell-laden hydrogel | IPF lung dECM | IPF fibroblast-laden 20 mg/mL IPF lung dECM, day 7 of culture | LLCT | Young's modulus | ~3.5 kPa | [23] |
| Human | Cell-laden hydrogel | Healthy lung dECM | Non-IPF fibroblast-laden 20 mg/mL healthy lung dECM hydrogels, day 14 of culture | LLCT | Young's modulus | ~4.25 kPa | [23] |
| Human | Cell-laden hydrogel | IPF lung dECM | IPF fibroblast-laden 20 mg/mL IPF lung dECM, day 14 of culture | LLCT | Young's modulus | ~3.6 kPa | [23] |
| Human | Hydrogel | Traut's reagent thiolated Lung dECM + PEG alpha methacrylate | Soft 7 % (wt/v) dECM-SH + 18% (wt/v) PEGaMA | Rheology | Young's modulus | 40 kPa | [47] |
| Human | Hydrogel | Traut's reagent thiolated Lung dECM + PEG alpha methacrylate | Soft 8 % (wt/v) dECM-SH + 17% (wt/v) PEGaMA | Rheology | Young's modulus | 15 kPa | [47] |
| Human | Hydrogel | Traut's reagent thiolated Lung dECM + PEG alpha methacrylate | Soft 8 % (wt/v) dECM-SH + 16% (wt/v) PEGaMA | Rheology | Young's modulus | ~ 10 kPa | [47] |
| Human | Hydrogel | Traut's reagent thiolated Lung dECM + PEG alpha methacrylate | Soft 10 % (wt/v) dECM-SH + 15% (wt/v) PEGaMA | Rheology | Young's modulus | ~ 5 kPa | [47] |
| Human | Hydrogel | Traut's reagent thiolated Lung dECM + PEG alpha methacrylate | Soft 12 % (wt/v) dECM-SH + 14% (wt/v) PEGaMA | Rheology | Young's modulus | ~ 1.25 kPa | [47] |
| Human | Hydrogel | Traut's reagent thiolated Lung dECM + PEG alpha methacrylate | Stiffened 6 % (wt/v) dECM-SH + 19% (wt/v) PEGaMA | Rheology | Young's modulus | ~ 90 kPa | [47] |
| Human | Hydrogel | Traut's reagent thiolated Lung dECM + PEG alpha methacrylate | Stiffened 7 % (wt/v) dECM-SH + 18% (wt/v) PEGaMA | Rheology | Young's modulus | ~ 70 kPa | [47] |
| Human | Hydrogel | Traut's reagent thiolated Lung dECM + PEG alpha methacrylate | Stiffened 8 % (wt/v) dECM-SH + 17% (wt/v) PEGaMA | Rheology | Young's modulus | 25 kPa | [47] |
| Human | Hydrogel | Traut's reagent thiolated Lung dECM + PEG alpha methacrylate | Stiffened 8 % (wt/v) dECM-SH + 16% (wt/v) PEGaMA | Rheology | Young's modulus | ~ 18 kPa | [47] |
| Human | Hydrogel | Traut's reagent thiolated Lung dECM + PEG alpha methacrylate | Stiffened 10 % (wt/v) dECM-SH + 15% (wt/v) PEGaMA | Rheology | Young's modulus | 15 kPa | [47] |
| Human | Hydrogel | Traut's reagent thiolated Lung dECM + PEG alpha methacrylate | Stiffened 12 % (wt/v) dECM-SH + 14% (wt/v) PEGaMA | Rheology | Young's modulus | 2.5 kPa | [47] |
| Murine | Hydrogel | Traut's reagent thiolated Lung dECM + poly(ethylene glycol) (PEG) alpha methacrylate | Soft 5 (mol%) dECM-SH + 250 mM DTT + 75 mM CGRDS peptide + 18.5% (wt/v) PEGαMA | Rheology | Young's modulus | ~20 kPa | [46] |
| Murine | Hydrogel | Traut's reagent thiolated Lung dECM + PEG alpha methacrylate | Soft 10 (mol%) dECM-SH + 250 mM DTT + 75 mM CGRDS peptide + 18.5% (wt/v) PEGαMA | Rheology | Young's modulus | ~11 kPa | [46] |
| Murine | Hydrogel | Traut's reagent thiolated Lung dECM + PEG alpha methacrylate | Soft 15% (mol%) dECM-SH + 250 mM DTT + 75 mM CGRDS peptide + 18.5% (wt/v) PEGαMA | Rheology | Young's modulus | ~5 kPa | [46] |
| Murine | Hydrogel | Traut's reagent thiolated Lung dECM + PEG alpha methacrylate | Soft 20 (mol%) dECM-SH + 250 mM DTT + 75 mM CGRDS peptide + 18.5% (wt/v) PEGαMA | Rheology | Young's modulus | ~ 2.5 kPa | [46] |
| Murine | Hydrogel | Traut's reagent thiolated Lung dECM + PEG alpha methacrylate | Soft 25 (mol%) dECM-SH + 250 mM DTT + 75 mM CGRDS peptide + 18.5% (wt/v) PEGαMA | Rheology | Young's modulus | ~ 1.25 kPa | [46] |
| Murine | Hydrogel | Traut's reagent thiolated Lung dECM + PEG alpha methacrylate | Stiffened 5 (mol%) dECM-SH + 250 mM DTT + 75 mM CGRDS peptide + 18.5% (wt/v) PEGαMA | Rheology | Young's modulus | ~ 25 kPa | [46] |
| Murine | Hydrogel | Traut's reagent thiolated Lung dECM + PEG alpha methacrylate | Stiffened 10 (mol%) dECM-SH + 250 mM DTT + 75 mM CGRDS peptide + 18.5% (wt/v) PEGαMA | Rheology | Young's modulus | ~ 20 kPa | [46] |
| Murine | Hydrogel | Traut's reagent thiolated Lung dECM + PEG alpha methacrylate | Stiffened 15% (mol%) dECM-SH + 250 mM DTT + 75 mM CGRDS peptide + 18.5% (wt/v) PEGαMA | Rheology | Young's modulus | ~ 10 kPa | [46] |
| Murine | Hydrogel | Traut's reagent thiolated Lung dECM + PEG alpha methacrylate | Stiffened 20 (mol%) dECM-SH + 250 mM DTT + 75 mM CGRDS peptide + 18.5% (wt/v) PEGαMA | Rheology | Young's modulus | ~ 4 kPa | [46] |
| Murine | Hydrogel | Traut's reagent thiolated Lung dECM + PEG alpha methacrylate | Stiffened 25 (mol%) dECM-SH + 250 mM DTT + 75 mM CGRDS peptide + 18.5% (wt/v) PEGαMA | Rheology | Young's modulus | ~ 2.5 kPa | [46] |
| Porcine | Hydrogel | Methacrylated lung dECM (dECM-MA) + methacrylated hyaluronic acid (HA/MA) | 10% dECM-MA/0.57% HA-MA diluted 0.40x | Rheology | Shear storage modulus | ~0.4 kPa | [65] |
| Porcine | Hydrogel | Lung dECM-MA + HA/MA | 10% dECM-MA/0.57% HA-MA diluted 0.45x | Rheology | Shear storage modulus | ~0.6 kPa | [65] |
| Porcine | Hydrogel | Lung dECM-MA + HA/MA | 10% dECM-MA/0.57% HA-MA diluted 0.50x | Rheology | Shear storage modulus | ~0.8 kPa | [65] |
| Porcine | Hydrogel | Lung dECM-MA + HA/MA | 10% dECM-MA/0.57% HA-MA diluted 0.55x | Rheology | Shear storage modulus | ~ 1 kPa | [65] |
| Porcine | Hydrogel | Lung dECM-MA + HA/MA | 10% dECM-MA/0.57% HA-MA diluted 0.60x | Rheology | Shear storage modulus | ~2.5 kPa | [65] |
| Porcine | Hydrogel | Lung dECM-MA + HA/MA | 10% dECM-MA/0.57% HA-MA diluted 0.65x | Rheology | Shear storage modulus | ~3 kPa | [65] |
| Porcine | Hydrogel | Lung dECM hydrogel | 10 mg/mL lung dECM | AFM | Young's modulus | ~225 Pa | [201] |
| Porcine | Hydrogel | Lung dECM hydrogel | 20 mg/mL lung dECM | AFM | Young's modulus | ~625 Pa | [201] |
| Porcine | Hydrogel | Lung dECMs | 8 (mg/mL) | Rheology | Storage modulus | 59.02 Pa | [206] |
| Porcine | Hydrogel | Lung dECMs | 6 (mg/mL) | Rheology | Storage modulus | 32.02 Pa | [206] |
| Porcine | Hydrogel | Lung dECMs | 4 (mg/mL) | Rheology | Storage modulus | 15.27 Pa | [206] |

^*^Stiffness values across studies should be interpreted with caution due to differences in testing protocols, including strain parameters.


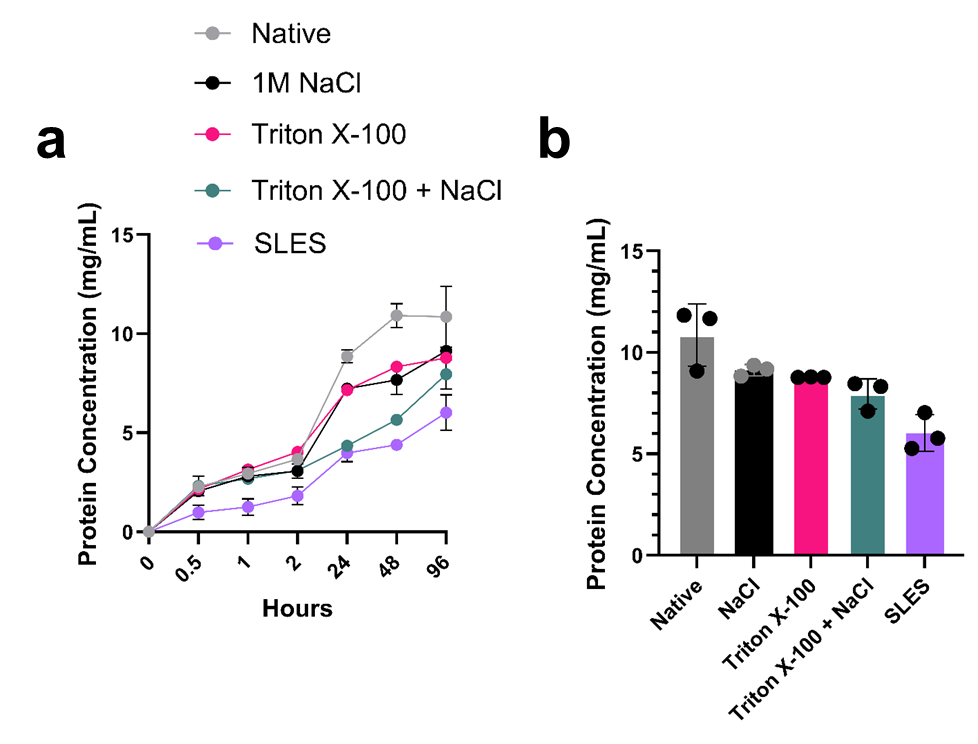


**Figure S5. Pepsin digestion of lung dECMs prepared through various decellularization methods over time. (A)** Protein concentration over time measured through NanoDrop One^C^ Spectrophotometer at wavelength A_280nm_. **(B)** Soluble protein concentration at end point of digestion (96 hours). N = 1. n = 3. All data is represented as mean ± SD.


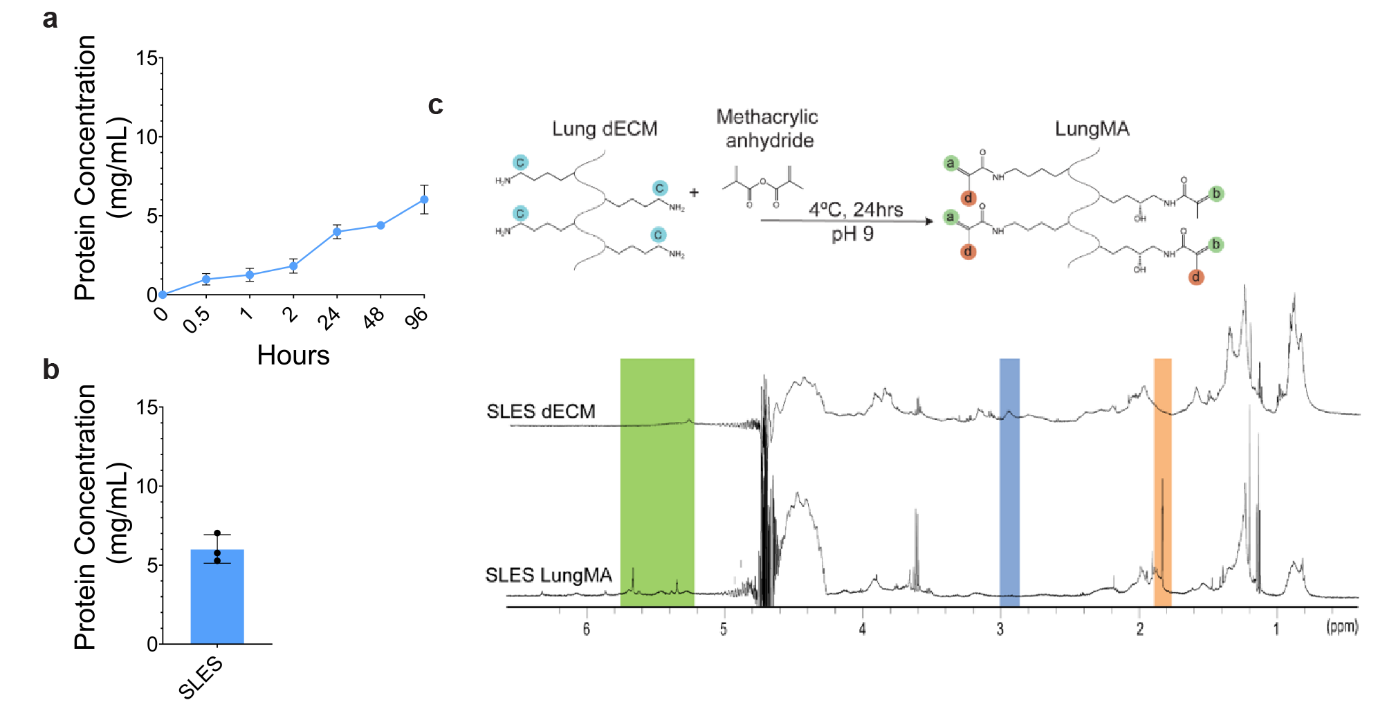


**Figure S6. ^1^H-NMR of SLES dECM and SLES LungMA.** New peaks in SLES LungMA at 5.8 ppm (a), 5.6 ppm (b), and 1.85 ppm (d), are indicative of newly grafted methacryloyl groups. Reduction in peak (c) at 2.99 ppm indicates loss of lysine-based amines due to functionalization.


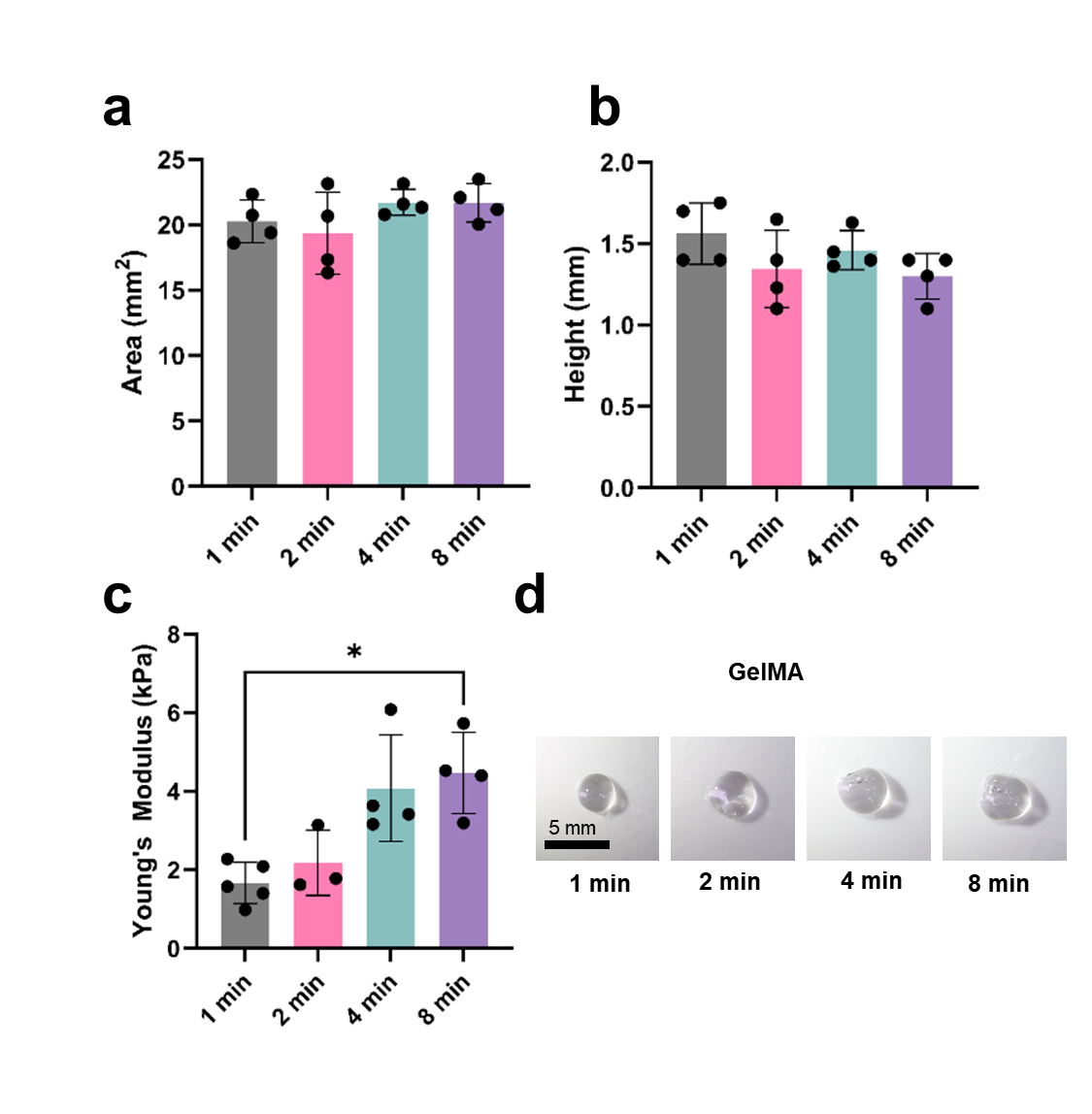


**Figure S7:** **Physicochemical properties of GelMA hydrogels.** Area **(A)**, area **(B)**, and Young’s modulus **(C)** of 5% (w/v) GelMA hydrogels crosslinked for 1 minute, 2 minutes, 4 minutes, and 8 minutes, show that hydrogel area and height is not significantly impacted by increased crosslinking time and Young’s modulus. Hydrogel height and Young’s modulus were determined via compression testing. **(D)** Stereoscopic images of GelMA hydrogels crosslinked with LAP under visible light (405 nm). Scale bar = 4 mm, magnification = 0.67x. N = 1. n = 4. One-way ANOVA was used for intergroup comparisons. All data is represented as mean ± SD. **p* < 0.1, ***p* < 0.01, ****p* < 0.001.


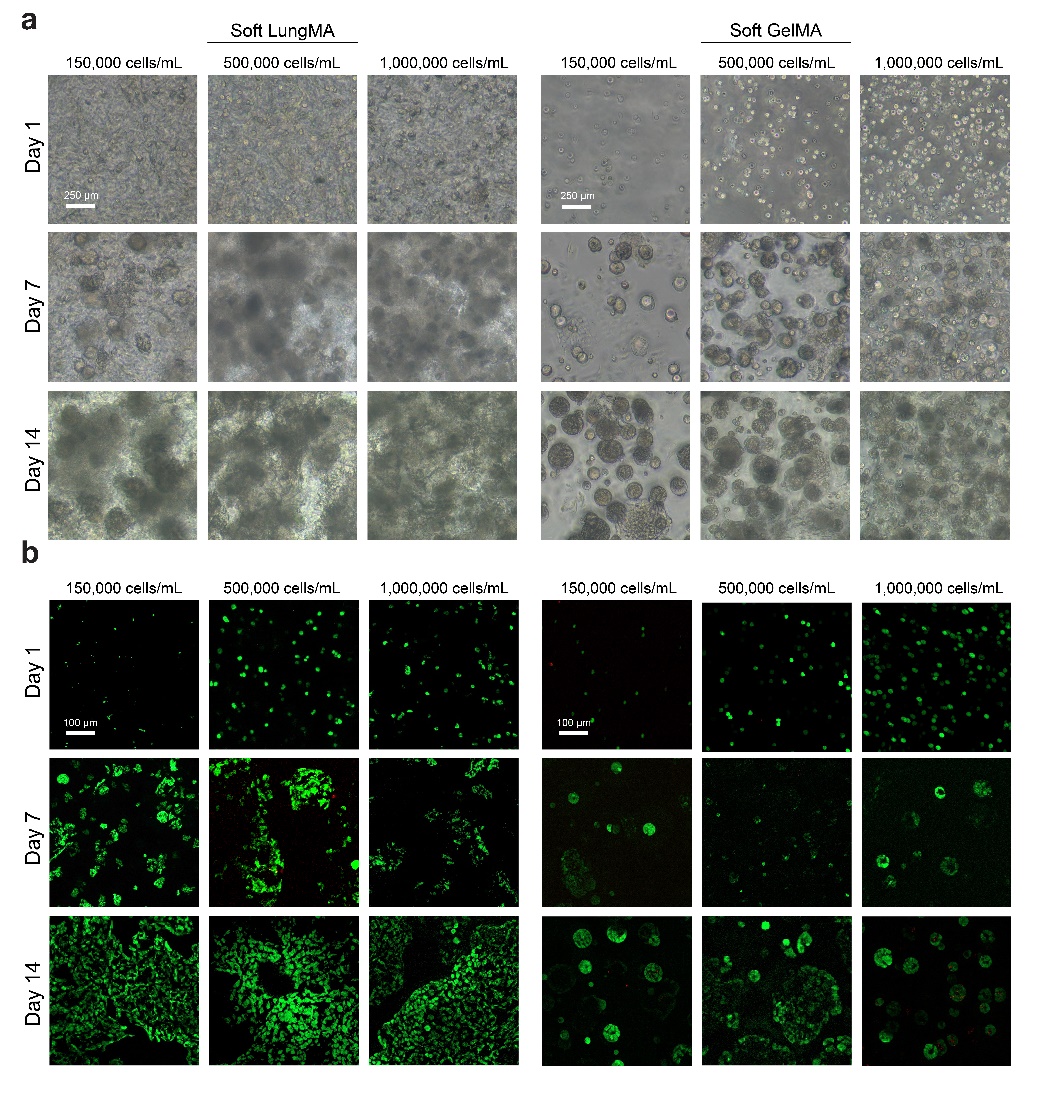


Figure S8. The effect of initial cell concentration on the growth characteristics of A549 lung cancer cells cultured in soft LungMA and GelMA hydrogels. (A) Brightfield microscopy of A549 cells. Objective = 4×. Scale bar = 250 µm. (B) Fluorescent microscopy of A549 cells. Objective = 10×. Scale = 100 µm. Fluorescein diacetate (FDA)/ propidium iodide (PI) staining was conducted to observe live (FDA, green) and dead (PI, red) cells. 1% (wt/v) LungMA hydrogels were crosslinked for 15 seconds in the presence of LAP photoinitiator to achieve a healthy lung mimicking Young’s modulus of 1 kPa. 5% (wt/v) GelMA hydrogels were crosslinked for 1 minute in the presence of LAP to achieve a healthy lung mimicking Young’s modulus of 1 kPa.


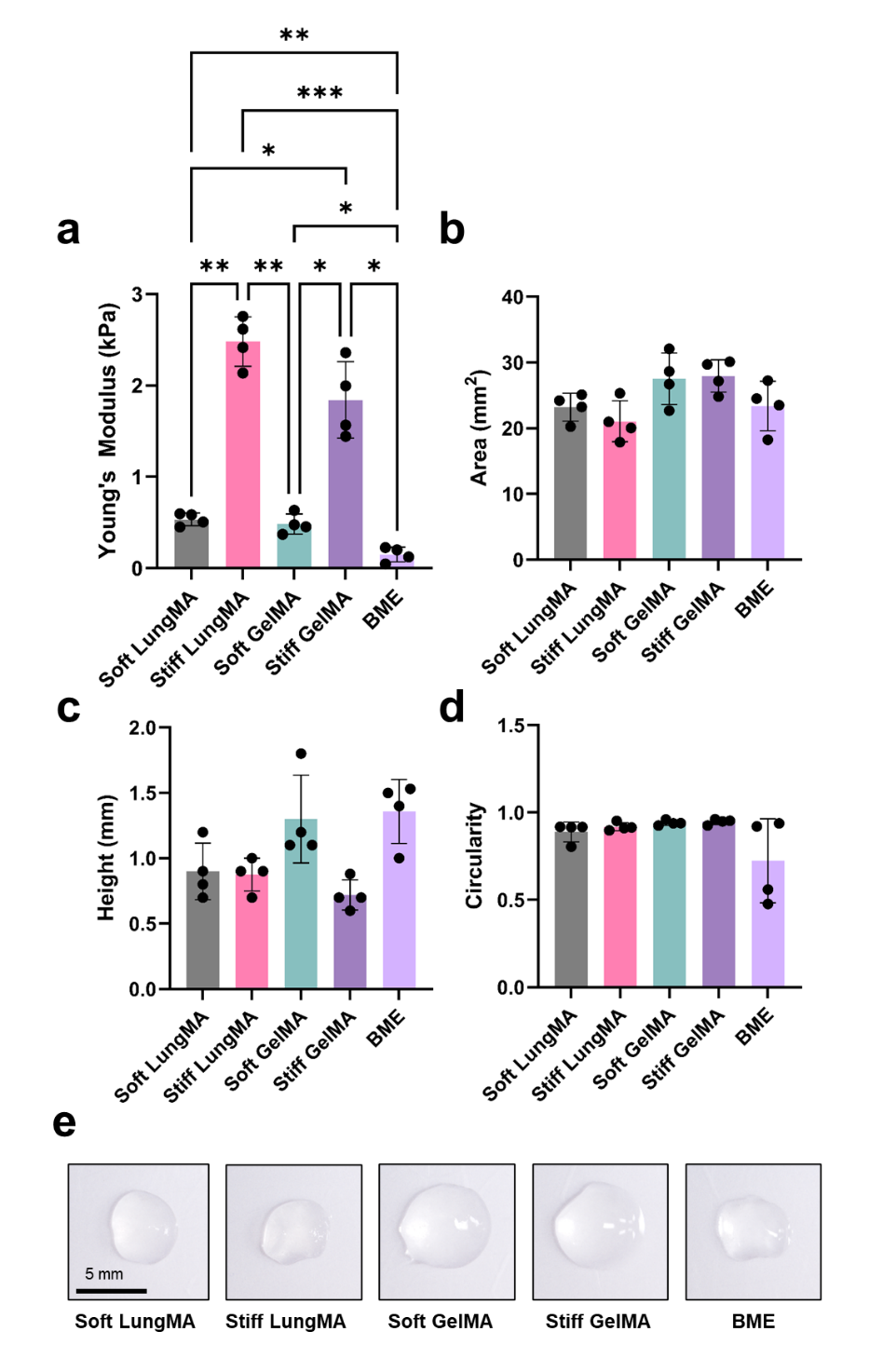


**Figure S9. Mechanical characterization of cell-laden soft LungMA, stiff LungMA, soft GelMA, stiff GelMA and BME hydrogels. (A)** Young’s modulus of A549-laden hydrogels on day 7 of culture determined via compression testing. **(B)** Area (mm^2^) of A549-laden hydrogels measured via ImageJ analysis of stereoscopic images of hydrogels. **(C)** Height (mm) of A549-laden hydrogels determined via compression testing. **(D)** Circularity of A549-laden hydrogels measured via ImageJ analysis of stereoscopic images of hydrogels. **(E)**. Representative brightfield images of A549-laden hydrogels prior to compression testing. Scale bar = 5 mm. N = 1. n = 4. All data is presented as mean ± SD. One-way ANOVA was used for intergroup comparisons. **p* < 0.1, ***p* < 0.01, ****p* < 0.001.

**
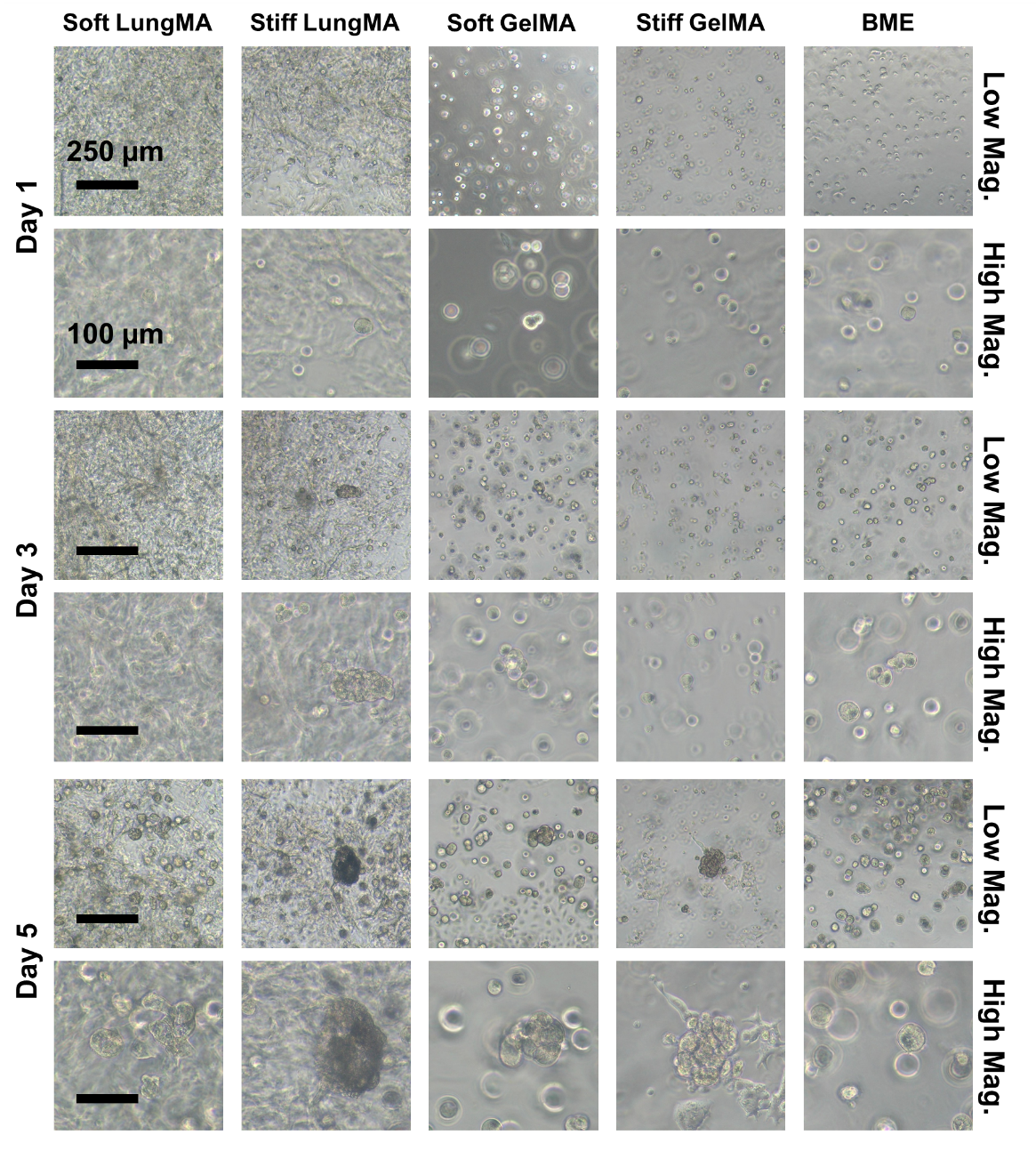
**

**Figure S10.1 Representative brightfield images of A549 cells cultured in 3D hydrogel matrices over time from day 1 to day 5.** A549s were seeded at a cell density of 500,00 cells/mL. Objective = 4×. Low magnification scale bar = 250 µm, high magnification scale bar = 100 µm.


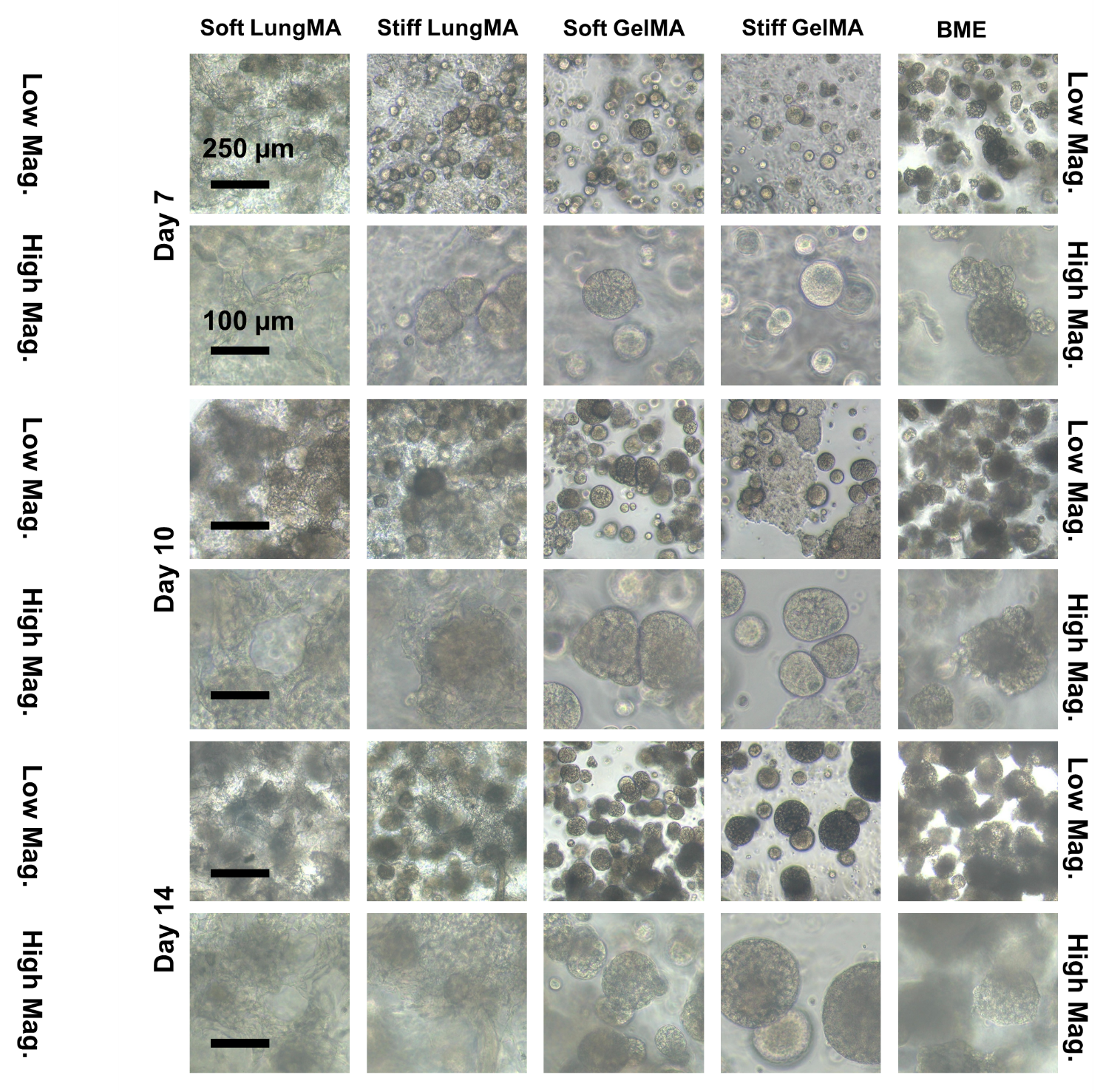


**Figure S10.2 Representative brightfield images of A549 cells cultured in 3D hydrogel matrices over time from day 7 to day 14.** A549s were seeded at a cell density of 500,00 cells/mL. Objective = 4×. Low magnification scale bar = 250 µm, high magnification scale bar = 100 µm.


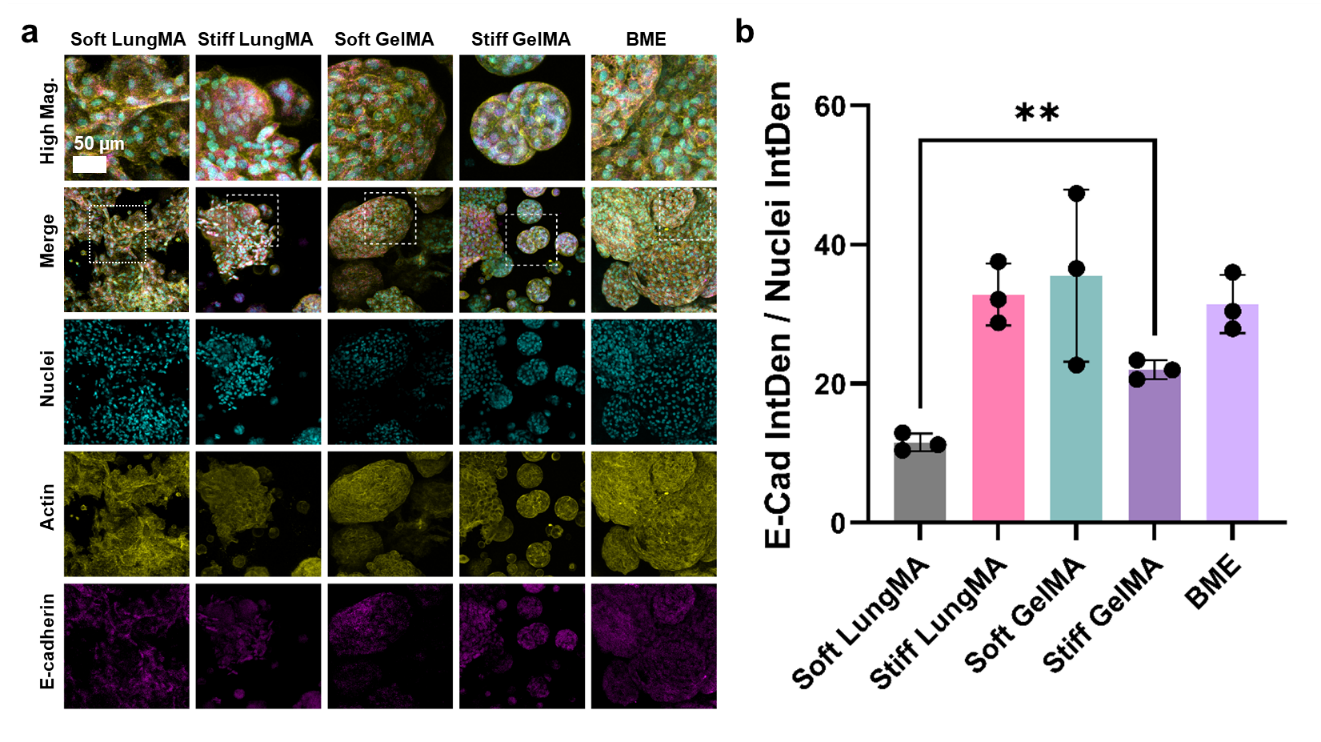


**Figure S11. Quantification of e-cadherin immunofluorescently stained A549-laden matrices. (A)** Representative images of e-cadherin stained A549s in hydrogels. A549 cells were cultured in soft (1 kPa) LungMA, stiff (>4 kPa) LungMA, soft GelMA, stiff GelMA, and Matrigel for 14 days, then fixed with 4% PFA. Fixed samples were permeabilized and fluorescently stained for nuclei (cyan), actin (yellow), and e-cadherin (magenta). Scale bar = 50 µm. **(B)** The integrated density (IntDen) of e-cadherin signals were normalized to the IntDen of the nuclei for each ROI. N = 1. n = 3. Mann Whitney test was used to analyze data. Data is represented as mean ± SD. ***p* < 0.01.


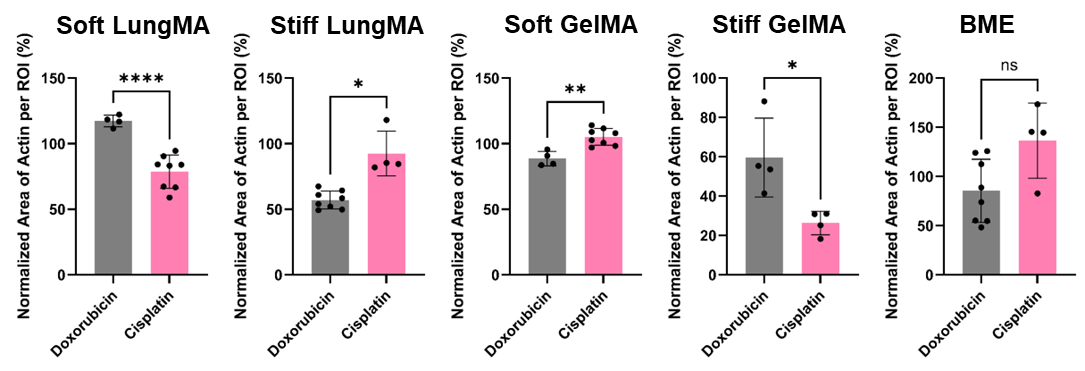


**Figure S12. The impact of drug treatment on area of actin per ROI.** Cultures were treated with mid-range chemotherapeutic agents and stained with phalloidin to observe actin distribution throughout matrices. The area of actin within treated samples was normalized to untreated samples. Area of actin per ROI (µm^2^) was determined through ImageJ analysis of phalloidin-stained cell actin fibers. N = 2. n = 4-8. Welch’s t-test was used to analyze data. All data is represented as mean ± SD. **p* < 0.1. ***p* < 0.01. *****p* < 0.0001.
